# Supplementary material for: A novel PLpro inhibitor improves outcomes in a pre-clinical model of long COVID
Source: Nat Commun. 2025 Apr 3;16:2900. doi: 10.1038/s41467-025-57905-4 (PMC11969009; doi:10.1038/s41467-025-57905-4)
Supplement: Supplementary file 1 — Supplementary Information [file 41467_2025_57905_MOESM1_ESM.pdf]

SUPPLEMENTARY FIGURES AND TABLES

A novel PLpro inhibitor improves outcomes in a pre-clinical model of long COVID

Stefanie M. Bader[1,2,^], Dale J. Calleja[1,2,^], Shane M. Devine[1,2,^,#], Nathan W. Kuchel[1,^], Bernadine G. C. Lu[1,2], Xinyu Wu[1,2], Richard W. Birkinshaw[1,2], Reet Bhandari[1,2], Katie Loi[1,2], Rohan Volpe[1,2], Yelena Khakham[1], Amanda E. Au[1,2], Timothy R. Blackmore[1,2], Liana Mackiewicz[1], Merle Dayton[1], Jan Schaefer[1,2], Lena Scherer[1], Angus T. Stock[1,2], James P. Cooney[1,2], Kael Schoffer[1,2], Ana Maluenda[1], Elizabeth A. Kleeman[1,3], Kathryn C. Davidson[1,2], Cody C. Allison[1,2], Gregor Ebert[1,2], Gong Chen[4], Kasiram Katneni[4], Theresa A. Klemm[1,2], Ueli Nachbur[1,2], Smitha Rose Georgy[5], Peter E. Czabotar[1,2], Anthony J. Hannan[3,6], Tracy L. Putoczki[1,2,7], Maria Tanzer[1,2], Marc Pellegrini[1,2,9], Bernhard C. Lechtenberg[1,2], Susan A. Charman[4], Melissa J. Call[1,2], Jeffrey P. Mitchell[1,2], Kym N. Lowes[1,2], Guillaume Lessene[1,2,8,#], Marcel Doerflinger[1,2,#], David Komander[1,2,#]

[1] Walter and Eliza Hall Institute of Medical Research, Parkville, Victoria, Australia.

[2] Department of Medical Biology, University of Melbourne, Melbourne, Australia.

[3] Florey Institute of Neuroscience and Mental Health, University of Melbourne, Parkville, Victoria, Australia.

[4] Centre for Drug Candidate Optimisation, Monash Institute of Pharmaceutical Sciences, Monash University, Parkville, Victoria, Australia

[5] Anatomic Pathology – Veterinary Biosciences, Melbourne Veterinary School, University of Melbourne, Werribee, Victoria, Australia

[6] Department of Anatomy and Physiology, University of Melbourne, Parkville, Victoria, Australia.

[7] Department of Surgery, University of Melbourne, Melbourne, Australia

[8] Department of Biochemistry and Pharmacology, University of Melbourne, Melbourne, Australia

[^] These authors contributed equally.

[9] Present address: Centenary Institute of Cancer Medicine and Cell Biology, Camperdown, NSW, Australia

[#] Corresponding authors. David Komander, dk@wehi.edu.au; Marcel Doerflinger, doerflinger.m@wehi.edu.au; Guillaume Lessene, glessene@wehi.edu.au; Shane M Devine, devine.s@wehi.edu.au.

Table of Contents

Supplementary Figures 3
Uncropped Gel 27
Medicinal Chemistry – General Experimental 28
Synthetic procedures 31
NMR Data 49
CYP Inhibition 61
Time dependent CYP3A4/5 inhibition 62
Mouse exposure parameters 63
Flow Cytometry Gating Strategy 64
Supplementary References 65

Supplementary Figures

a

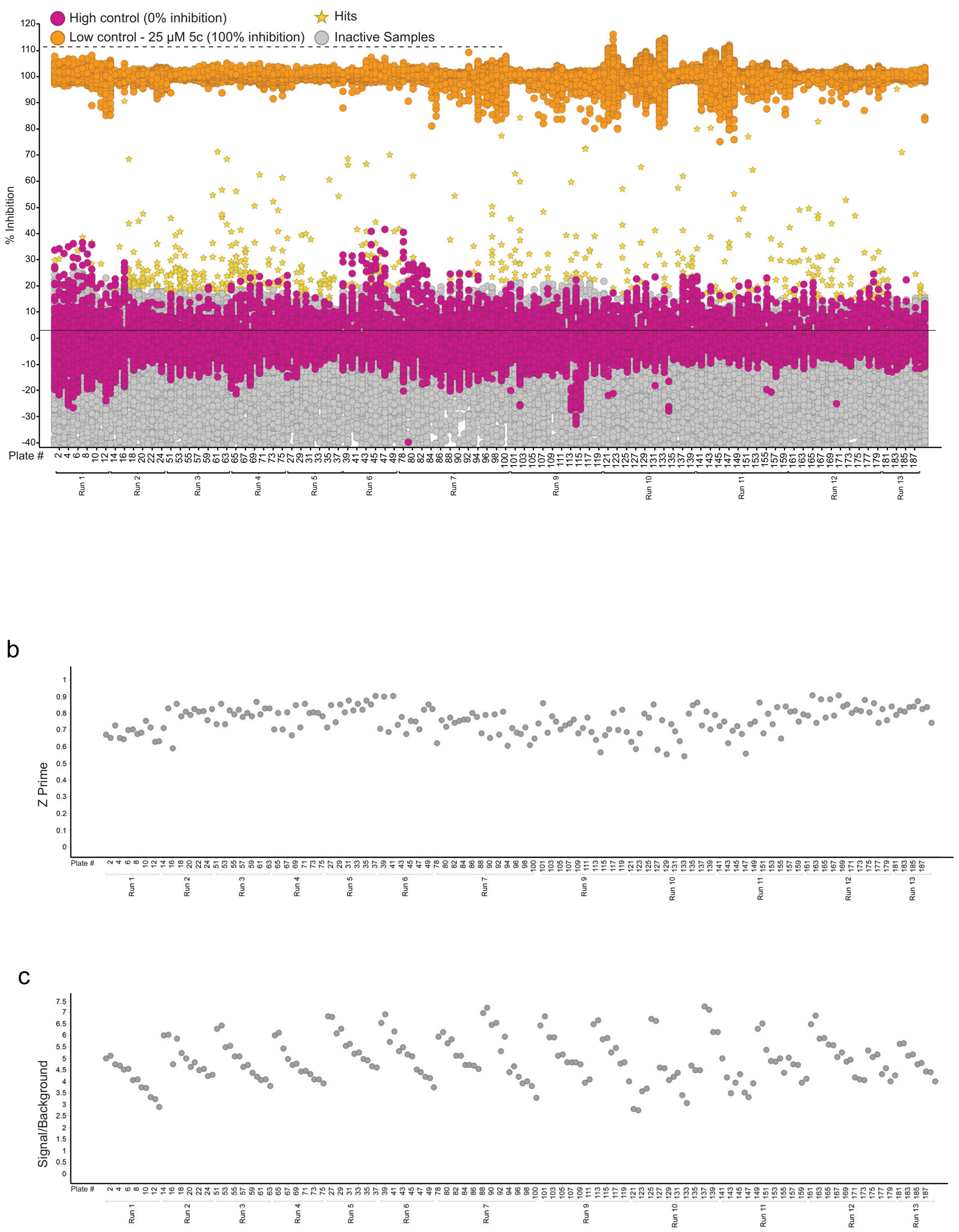

b

c

Supplementary Figure 1. High Throughput Screen of SARS-CoV-2 PLpro

Results from the primary screen by plate number grouped by run number (#). The UbRh assay (see **Fig. 1a**) assay was used to identify inhibitors of PLpro (see **Methods**). **(a)** A total of 412,644 compounds were screened at a single concentration. Hits were identified based on the criteria that they were 3 standard deviations above the mean of the high control (0% inhibition). **(b)** Z Prime (Z') analysis of the primary screen by plate number. All plates met quality criteria of Z' > 0.5. **(c)** Signal/background (S/B) analysis of the primary screen by plate number. All plates met the quality control criteria (S/B > 3).

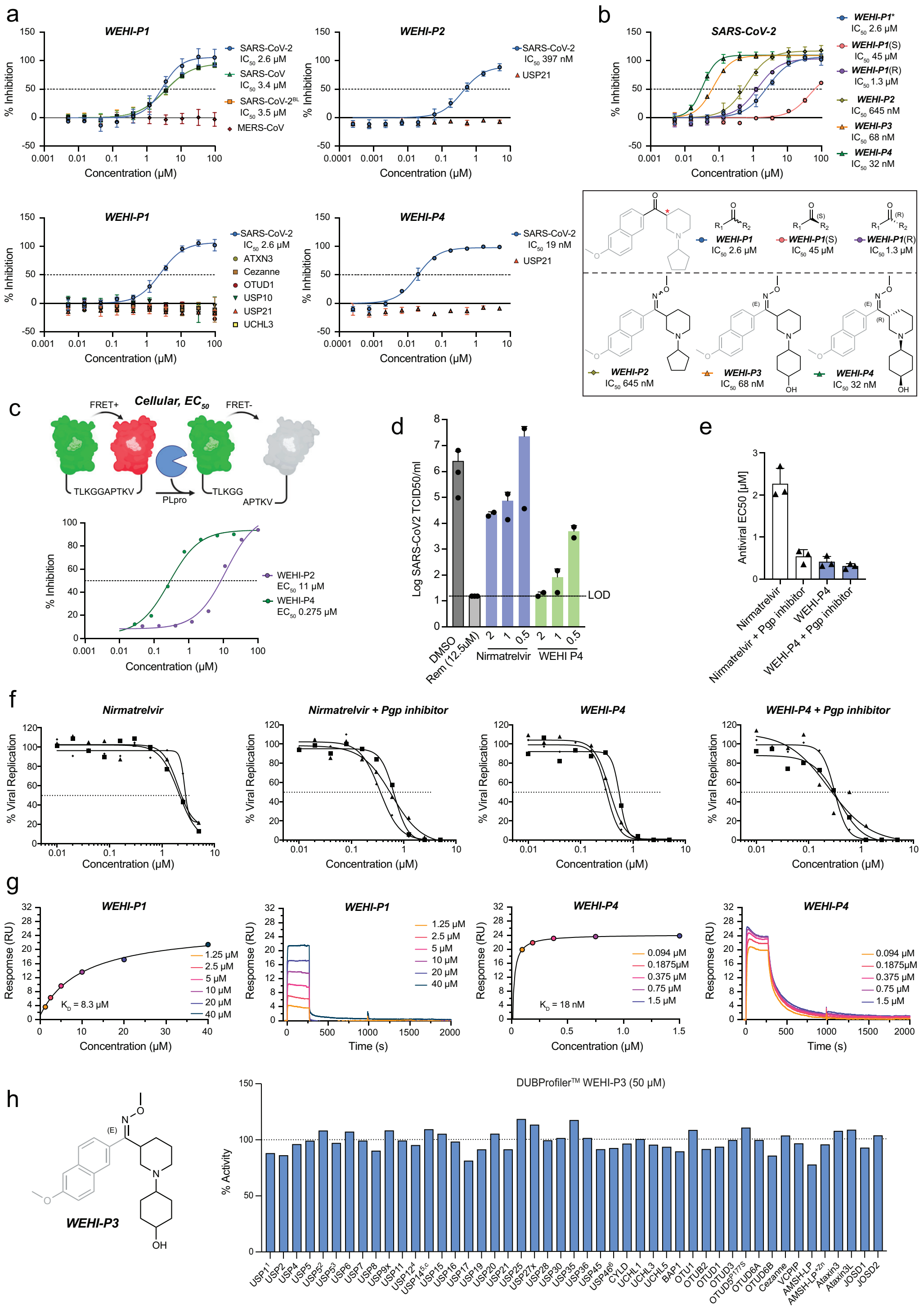
a
WEHI-P1
SARS-CoV-2 IC50 2.6 µM
SARS-CoV IC50 3.4 µM
SARS-CoV-2BL IC50 3.5 µM
MERS-CoV
WEHI-P2
SARS-CoV-2 IC50 397 nM
USP21
WEHI-P1
SARS-CoV-2 IC50 2.6 µM
ATXN3
Cezanne
OTUD1
USP10
USP21
UCHL3
WEHI-P4
SARS-CoV-2 IC50 19 nM
USP21
% Inhibition
Concentration (µM)
b
SARS-CoV-2
WEHI-P1* IC50 2.6 µM
WEHI-P1(S) IC50 45 µM
WEHI-P1(R) IC50 1.3 µM
WEHI-P2 IC50 645 nM
WEHI-P3 IC50 68 nM
WEHI-P4 IC50 32 nM
WEHI-P1 IC50 2.6 µM
WEHI-P1(S) IC50 45 µM
WEHI-P1(R) IC50 1.3 µM
WEHI-P2 IC50 645 nM
WEHI-P3 IC50 68 nM
WEHI-P4 IC50 32 nM
c
Cellular, EC50
FRET+
FRET-
TLKGGAPTKV
PLpro
TLKGG
APTKV
WEHI-P2 EC50 11 µM
WEHI-P4 EC50 0.275 µM
% Inhibition
Concentration (µM)
d
Log SARS-CoV2 TCID50/ml
LOD
DMSO
Rem (12.5uM)
Nirmatrelvir
WEHI P4
e
Antiviral EC50 [µM]
Nirmatrelvir
Nirmatrelvir + Pgp inhibitor
WEHI-P4
WEHI-P4 + Pgp inhibitor
f
Nirmatrelvir
Nirmatrelvir + Pgp inhibitor
WEHI-P4
WEHI-P4 + Pgp inhibitor
% Viral Replication
Concentration (µM)
g
WEHI-P1
KD = 8.3 µM
1.25 µM
2.5 µM
5 µM
10 µM
20 µM
40 µM
WEHI-P4
KD = 18 nM
0.094 µM
0.1875µM
0.375 µM
0.75 µM
1.5 µM
Response (RU)
Concentration (µM)
Time (s)
h
WEHI-P3
DUBProfiler™ WEHI-P3 (50 µM)
% Activity
USP1 USP2 USP4 USP5 USP52 USP53 USP6 USP7 USP8 USP9x USP11 USP124 USP14 USP15 USP16 USP17 USP19 USP20 USP21 USP25 USP27x USP28 USP30 USP35 USP36 USP45 USP46 CYLD UCHL1 UCHL3 UCHL5 BAP1 OTU1 OTUB2 OTUD1 OTUD3 OTUD5 OTUD6A OTUD6B Cezanne VCPIP AMSH-LP AMSH-LP Ataxin3 Ataxin3L JOSD1 JOSD2

Supplementary Figure 2. Assay pipeline to assess compound activity of the *WEHI-P* series.

(a-b) The Ubiquitin Rhodamine110 assay (see **Fig. 1a**) was used to assess the activity of all compounds against PLpro variants and control DUBs. Biochemical IC_{50} is displayed as the mean ± SD of n = 2-4 independent experiments each with technical duplicates. A representative curve for each is shown. ***WEHI-P1*** upper panel, n=3 for SARS-CoV-2, n=4 for SARS-CoV and n=2 for the remaining DUBs; ***WEHI-P1*** lower panel, n=4 for all PLpro variants; ***WEHI-P2***, n=4 and ***WEHI-P4***, n=2. See **Methods** for further assay details. **(b)** The UbRh assay was used to assess the activity of ***WEHI-P1*** and its enantiomeric pairs ***WEHI-P1(S)*** and ***WEHI-P1(R)***, ***WEHI-P2***, ***WEHI-P3*** and ***WEHI-P4***. **(c)** Cellular FRET biosensor assay[1] used for measuring in cell activity of PLpro inhibitors. Cleavage of the biosensor linker by PLpro releases the mClover-(green)/mRuby (red) FRET pair and induces a loss in fluorescent signal. ***WEHI-P1*** exhibited low activity in this assay (>20 µM, data not shown). Data is displayed as the mean of two independent experiments (n=2) performed with technical triplicate in each experiment.

(d) Time-course of TCID50 analysis of infectious virus for ***WEHI-P4*** and nirmatrelvir. Vero cells were infected with SARS-CoV-2 containing supernatant obtained from infected Calu-3 cells and treated as shown (see Methods). Data are from 2 independent experiments (3 experiments for DMSO and remdesivir (Rem) controls), with 6 technical replicates per experiment, bars represent the mean TCID50 value with error bars showing SD. **(e)** An antiviral plaque assay was used to assess the activity of ***WEHI-P*** series on viral replication. ***WEHI-P1*** to ***WEHI-P3*** showed low activity (>5 µM, data not

shown). Nirmatrelvir was used to benchmark ***WEHI-P4*** with or without the Pgp inhibitor CP100356 (2 μM). Data is displayed as the mean of experiments performed in triplicate with error bars showing SD. **(f)** Corresponding inhibition curves. **(g)** SPR was used to measure direct binding of compound from the ***WEHI-P*** series to PLpro. *Top*, fitted binding curve; *bottom*, sensorgram data. K_D shown is the mean of experiments performed in technical duplicate. Representative graphs are shown. **(h)** Inhibitor ***WEHI-P3*** was tested at the DUBprofiler platform at Ubiquigent (Dundee, UK) assessing inhibitory potential against humans DUBs at 50 μM inhibitor concentration. USP1[1], USP1 assay performed in the presence of UAF1; USP5[2,3], USP5 assay performed with addition of ubiquitin at K_D (USP5[2]) *or* B_{max} (USP5[3]); USP12[4]/USP46[6] indicates USP12/USP46 assays performed in the presence of modulating enzymes UAF1/WDR20; USP14[5] indicates assay performed in the presence of proteasome-vinyl sulfone at K_D; USP14[c] indicates the proteasome-vinyl sulfone control showed no activity in the absence of USP14. **Supplementary Fig. 2c** - Created in BioRender. Calleja, D. (2025) https://BioRender.com/s97k322

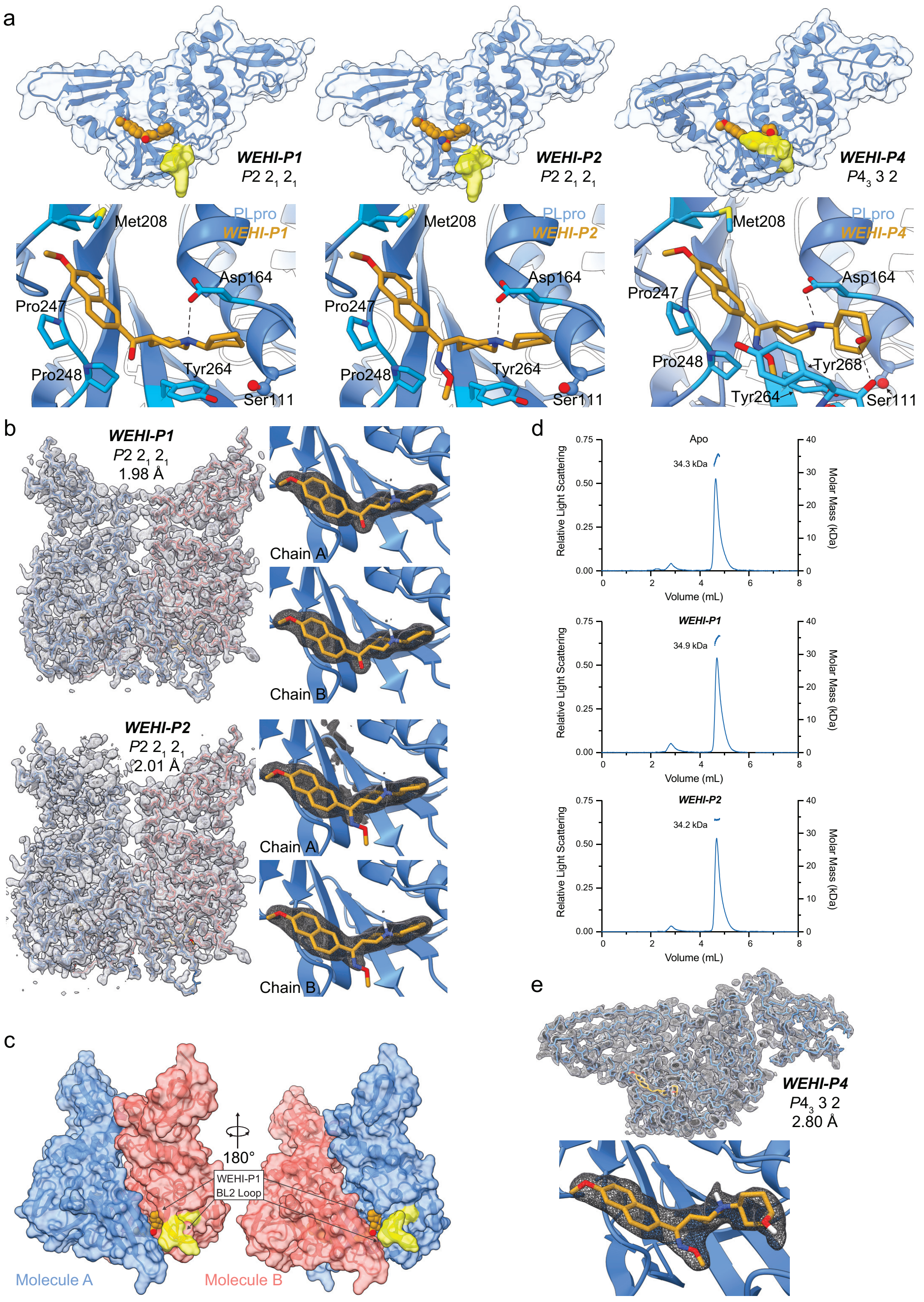
a
WEHI-P1
P2 2$_1$ 2$_1$
WEHI-P2
P2 2$_1$ 2$_1$
WEHI-P4
P4$_3$ 3 2
PLpro
WEHI-P1
Met208
Asp164
Pro247
Pro248
Tyr264
Ser111
PLpro
WEHI-P2
Met208
Asp164
Pro247
Pro248
Tyr264
Ser111
PLpro
WEHI-P4
Met208
Asp164
Pro247
Pro248
Tyr268
Tyr264
Ser111
b
WEHI-P1
P2 2$_1$ 2$_1$
1.98 Å
Chain A
Chain B
WEHI-P2
P2 2$_1$ 2$_1$
2.01 Å
Chain A
Chain B
c
180°
WEHI-P1
BL2 Loop
Molecule A
Molecule B
d
Apo
34.3 kDa
Relative Light Scattering
Molar Mass (kDa)
Volume (mL)
WEHI-P1
34.9 kDa
Relative Light Scattering
Molar Mass (kDa)
Volume (mL)
WEHI-P2
34.2 kDa
Relative Light Scattering
Molar Mass (kDa)
Volume (mL)
e
WEHI-P4
P4$_3$ 3 2
2.80 Å

Supplementary Figure 3. Structural insights of the WEHI-P series.

(**a**) Crystal structure of SARS-CoV-2 PLpro in complex with ***WEHI-P1***, ***WEHI-P2*** and ***WEHI-P4*** were solved in two different space groups. The BL2 region of PLpro is coloured yellow. In ***WEHI-P1*** and ***WEHI-P2*** complex structures in space group $P2\ 2_1\ 2_1$, the BL2 loop does not contribute to compound binding but is involved in a crystal contact. In ***WEHI-P4*** the BL2 region is in the 'closed' conformation to contact the compound. A zoomed view of PLpro bound to each compound is also shown below. **(b)** 2|Fo| – |Fc| electron density contoured at 1 s of the contents of the asymmetric unit. Insets show the compound binding sites with ligand density. For ***WEHI-P2***, the oxime group remained unresolved. **(c)** Surface representation of the protein chains in asymmetric unit of the $P2\ 2_1\ 2_1$ crystal setting (***WEHI-P1***, ***-P2***). Compounds were wedged between molecules. For this reason, we determined compound structures in another space group. **(d)** SEC–MALS analysis of SARS-CoV-2 PLpro apo form or in the presence of 100 µM ***WEHI-P1*** or ***WEHI-P2***. Absorbance was measured at a wavelength of 280 nm. Relative light scattering (Rayleigh Ratio) and calculated molecular mass over each peak is shown, with the observed molecular weights indicated in the bottom left. SEC–MALS experiments were performed once each with $PLpro^{WT}$ and $PLpro^{C111S}$, which displayed identical behaviour (graphs for $PLpro^{WT}$ are shown). **(e)** 2|Fo| – |Fc| electron density map at 1 s, of the asymmetric content of the ***WEHI-P4*** complex in space group $P4_3\ 3\ 2$, with an overall view (top) and showing the ligand density (below). In this structure, the oxime is resolved clearly, and there are no crystal contacts affecting the compound binding site.

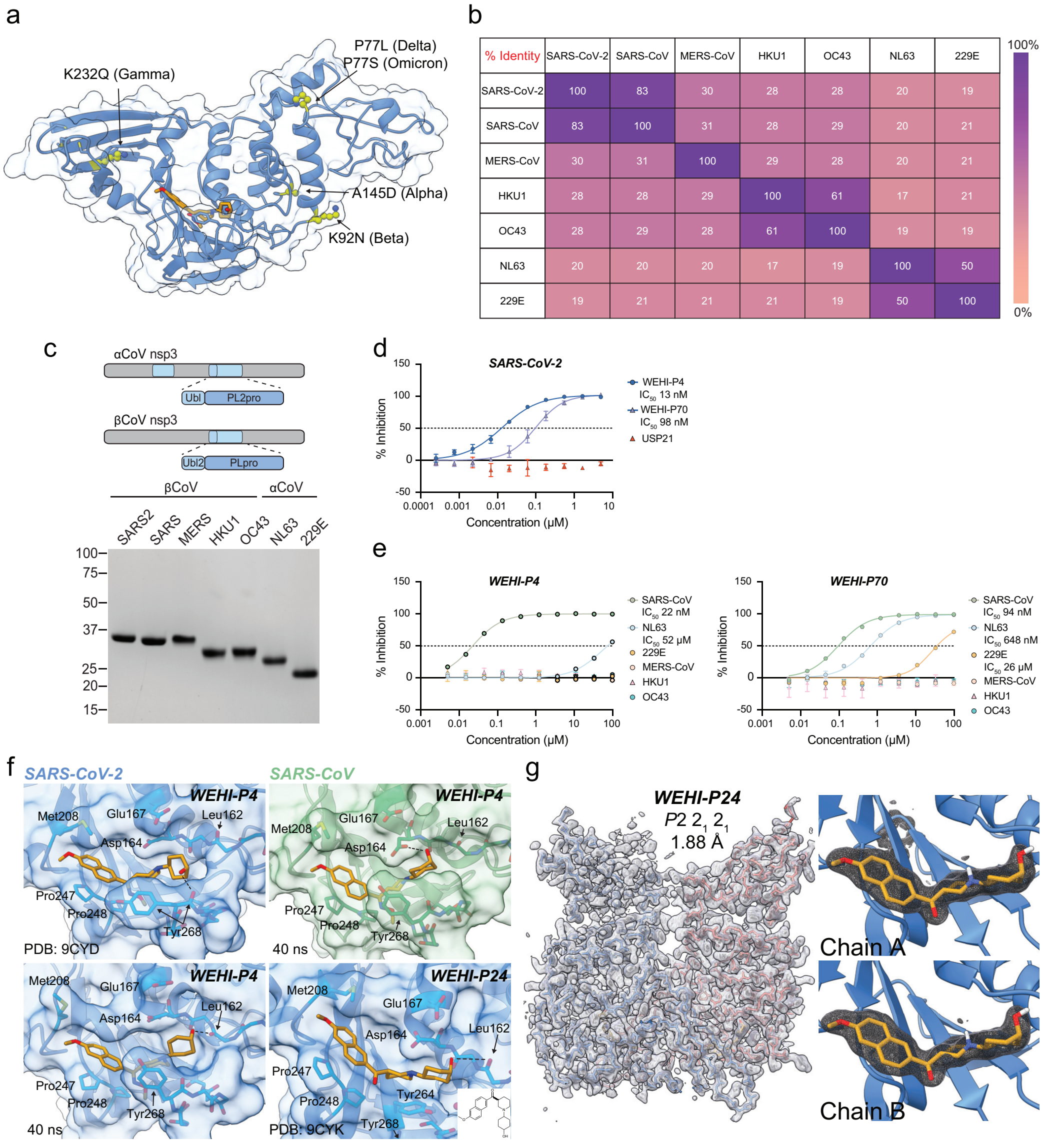
a
K232Q (Gamma)
P77L (Delta)
P77S (Omicron)
A145D (Alpha)
K92N (Beta)
b
% Identity
SARS-CoV-2 SARS-CoV MERS-CoV HKU1 OC43 NL63 229E
SARS-CoV-2 100 83 30 28 28 20 19
SARS-CoV 83 100 31 28 29 20 21
MERS-CoV 30 31 100 29 28 20 21
HKU1 28 28 29 100 61 17 21
OC43 28 29 28 61 100 19 19
NL63 20 20 20 17 19 100 50
229E 19 21 21 21 19 50 100
100%
0%
c
αCoV nsp3
Ubl PL2pro
βCoV nsp3
Ubl2 PLpro
βCoV αCoV
SARS2 SARS MERS HKU1 OC43 NL63 229E
100 75 50 37 25 20 15
d
SARS-CoV-2
WEHI-P4 IC50 13 nM
WEHI-P70 IC50 98 nM
USP21
% Inhibition
Concentration (μM)
e
WEHI-P4
SARS-CoV IC50 22 nM
NL63 IC50 52 μM
229E
MERS-CoV
HKU1
OC43
WEHI-P70
SARS-CoV IC50 94 nM
NL63 IC50 648 nM
229E IC50 26 μM
MERS-CoV
HKU1
OC43
% Inhibition
Concentration (μM)
f
SARS-CoV-2
SARS-CoV
WEHI-P4
WEHI-P24
Met208 Glu167 Leu162 Asp164 Pro247 Pro248 Tyr268 Tyr264
PDB: 9CYD
40 ns
40 ns
PDB: 9CYK
g
WEHI-P24
P2 2₁ 2₁
1.88 Å
Chain A
Chain B

Supplementary Figure 4. Molecular Insights into pan-CoV activity of *WEHI-P70.*

(a) SARS-CoV-2 PLpro bound to ***WEHI-P4*** (**Fig. 1c**), highlighting mutations from currently circulating SARS-CoV-2 variants of concern. All mutations are remote from compound binding sites. **(b)** Sequence identity matrix of the PLpro and PL2pro domains from the seven human transmissible coronaviruses, see sequence alignment in **Supplementary Fig 5**. **(c)** PLpro (bCoVs) and PL2pro (aCoVs) domains were expressed and purified to homogeneity for use in UbRh biochemical activity assays, against ***WEHI-P*** series compounds (see **Methods** for constructs). **(d-e)** Biochemical IC_{50} is displayed as the mean of n = 2 independent experiments each with technical duplicates. A representative curve for each is shown. See **Methods** for further assay details **(d)** Biochemical IC_{50} comparing ***WEHI-P4*** and ***WEHI-P70*** in inhibiting SARS-CoV-2 PLpro. Both compounds remain selective over USP21. USP21 data for ***WEHI-P70*** is shown (see **Supplementary Figure 2a** for ***WEHI-P4***). **(e)** Biochemical IC_{50}s comparing ***WEHI-P4*** and ***WEHI-P70*** in inhibiting PLpro from different CoVs. **(f)** Comparison of experimental structures of ***WEHI-P4*** and ***WEHI-P24*** bound to SARS-CoV-2 PLpro with 40 ns molecular dynamics simulations of docking ***WEHI-P4*** into SARS-CoV-2 and SARS-CoV PLpro. Key residues are noted. The cyclohexanol forms a H-bond with either the backbone of Leu162 or with that of Tyr268. **(g)** A further compound structure of PLpro bound to ***WEHI-P24*** was determined at 1.88 Å resolution in $P2\ 2_1\ 2_1$ (see **Supplementary Table 1**). 2|Fo| – |Fc| electron density at 1 s covers the protein chains (*left*) and ligand binding site (*right*).

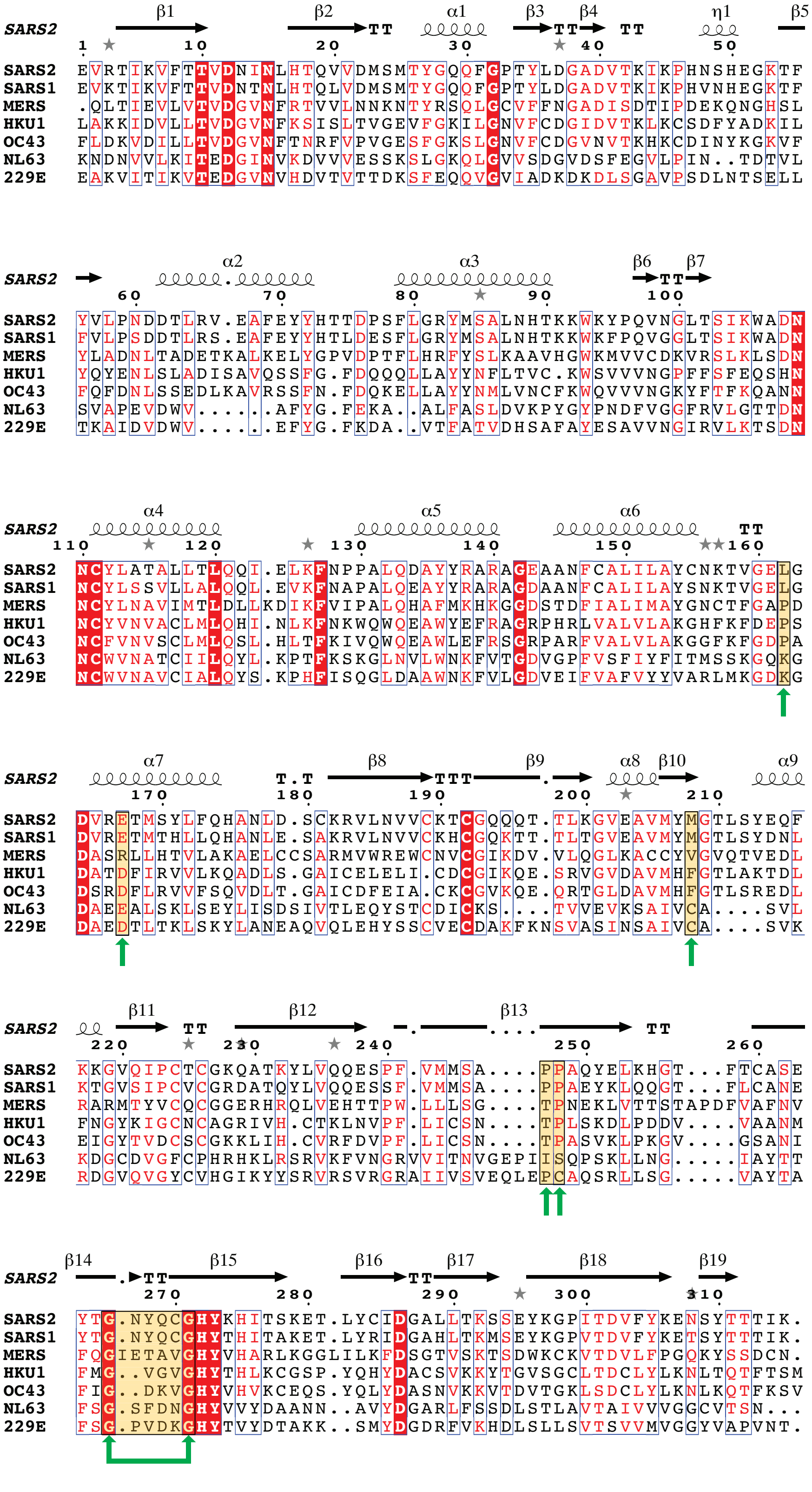
SARS2 EVRTIKVFTTVDNINLHTQVVDMSMTYGQQFGPTYLDGADVTKIKPHNSHEGKTF
SARS1 EVKTIKVFTTVDNTNLHTQLVDMSMTYGQQFGPTYLDGADVTKIKPHVNHEGKTF
MERS .QLTIEVLVTVDGVNFRTVVLNNKNTYRSQLGCVFFNGADISDTIPDEKQNGHSL
HKU1 LAKKIDVLLTVDGVNFKSISLTVGEVFGKILGNVFCDGIDVTKLKCSDFYADKIL
OC43 FLDKVDILLTVDGVNFTNRFVPVGESFGKSLGNVFCDGVNVTKHKCDINYKGKVF
NL63 KNDNVVLKITEDGINVKDVVVESSKSLGKQLGVVSDGVDSFEGVLPIN..TDTVL
229E EAKVITIKVTEDGVNVHDVTVTTDKSFEQQVGVIADKDKDLSGAVPSDLNTSELL
SARS2 YVLPNDDTLRV.EAFEYYHTTDPSFLGRYMSALNHTKKWKYPQVNGLTSIKWADN
SARS1 FVLPSDDTLRS.EAFEYYHTLDESFLGRYMSALNHTKKWKFPQVGGLTSIKWADN
MERS YLADNLTADETKALKELYGPVDPTFLHRFYSLKAAVHGWKMVVCDKVRSLKLSDN
HKU1 YQYENLSLADISAVQSSFG.FDQQQLLAYYNFLTVC.KWSVVVNGPFFSFEQSHN
OC43 FQFDNLSSEDLKAVRSSFN.FDQKELLAYYNMLVNCFKWQVVVNGKYFTFKQANN
NL63 SVAPEVDWV.....AFYG.FEKA..ALFASLDVKPYGYPNDFVGGFRVLGTTDN
229E TKAIDVDWV.....EFYG.FKDA..VTFATVDHSAFAYESAVVNGIRVLKTSDN
SARS2 NCYLATALLTLQQI.ELKFNPPALQDAYYRARAGEAANFCALILAYCNKTVGELG
SARS1 NCYLSSVLLALQQL.EVKFNAPALQEAYYRARAGDAANFCALILAYSNKTVGELG
MERS NCYLNAVIMTLDLLKDIKFVIPALQHAFMKHKGGDSTDFIALIMAYGNCTFGAPD
HKU1 NCYVNVACLMLQHI.NLKFNKWQWQEAWYEFRAGRPHRLVALVLAKGHFKFDEPS
OC43 NCFVNVSCLMLQSL.HLTFKIVQWQEAWLEFRSGRPARFVALVLAKGGFKFGDPA
NL63 NCWVNATCIILQYL.KPTFKSKGLNVLWNKFVTGDVGPFVSFIYFITMSSKGQKG
229E NCWVNAVCIALQYS.KPHFISQGLDAAWNKFVLGDVEIFVAFVYYVARLMKGDKG
SARS2 DVRETMSYLFQHANLD.SCKRVLNVVCKTCGQQQT.TLKGVEAVMYMGTLSYEQF
SARS1 DVRETMTHLLQHANLE.SAKRVLNVVCKHCGQKTT.TLTGVEAVMYMGTLSYDNL
MERS DASRLLHTVLAKAELCCSARMVWREWCNVCGIKDV.VLQGLKACCYVGVQTVEDL
HKU1 DATDFIRVVLKQADLS.GAICELELI.CDCGIKQE.SRVGVDAVMHFGTLAKTDL
OC43 DSRDFLRVVFSQVDLT.GAICDFEIA.CKCGVKQE.QRTGLDAVMHFGTLSREDL
NL63 DAEEALSKLSEYLISDSIVTLEQYSTCDICKS....TVVEVKSAIVCA....SVL
229E DAEDTLTKLSKYLANEAQVQLEHYSSCVECDAKFKNSVASINSAIVCA....SVK
SARS2 KKGVQIPCTCGKQATKYLVQQESPF.VMMSA....PPAQYELKHGT...FTCASE
SARS1 KTGVSIPCVCGRDATQYLVQQESSF.VMMSA....PPAEYKLQQGT...FLCANE
MERS RARMTYVCQCGGERHRQLVEHTTPW.LLLSG....TPNEKLVTTSTAPDFVAFNV
HKU1 FNGYKIGCNCAGRIVH.CTKLNVPF.LICSN....TPLSKDLPDDV....VAANM
OC43 EIGYTVDCSCGKKLIH.CVRFDVPF.LICSN....TPASVKLPKGV....GSANI
NL63 KDGCDVGFCPHRHKLRSRVKFVNGRVVITNVGEPIISQPSKLLNG.....IAYTT
229E RDGVQVGYCVHGIKYYSRVRSVRGRAIIVSVEQLEPCAQSRLLSG.....VAYTA
SARS2 YTG.NYQCGHYKHITSKET.LYCIDGALLTKSSEYKGPITDVFYKENSYTTTIK.
SARS1 YTG.NYQCGHYTHITAKET.LYRIDGAHLTKMSEYKGPVTDVFYKETSYTTTIK.
MERS FQGIETAVGHYVHARLKGGLILKFDSGTVSKTSDWKCKVTDVLFPGQKYSSDCN.
HKU1 FMG..VGVGHYTHLKCGSP.YQHYDACSVKKYTGVSGCLTDCLYLKNLTQTFTSM
OC43 FIG..DKVGHYVHVKCEQS.YQLYDASNVKKVTDVTGKLSDCLYLKNLKQTFKSV
NL63 FSG.SFDNGHYVVYDAANN..AVYDGARLFSSDLSTLAVTAIVVGGCVTSN...
229E FSG.PVDKGHYTVYDTAKK..SMYDGDRFVKHDLSLLSVTSVVMVGGYVAPVNT.

Supplementary Figure 5. Multiple Sequence Alignment of PLpro domains.

Sequence alignment of the PLpro domains from the seven human transmissible coronaviruses. For aCoVs 229E and NL63, the PL2pro domain is evolutionarily related and serves a DUB domain[2]. Key residues found in the inhibitor binding sites for ***WEHI-P*** series compounds are highlighted and indicated with green arrows.

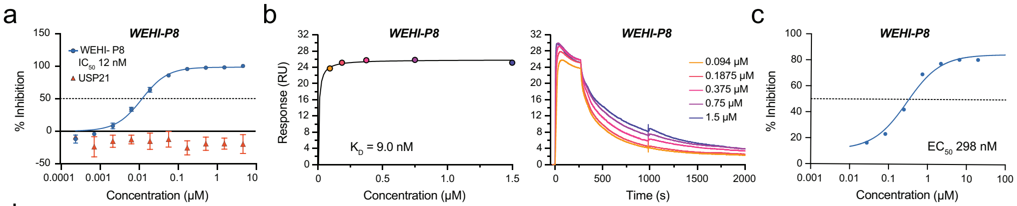

d

In vitro PK	WEHI-P8
Mouse microsomes	$t_{1/2}$ = 41.5 min<br>CL_{int} = 33.4 µL/min/mg protein
Human microsomes	$t_{1/2}$ = 144 min<br>CL_{int} = 9.7 µL/min/mg protein
Mouse hepatocytes	$t_{1/2}$ = 82.6 min<br>CL_{int} = 16.8 µL/min/10^6 cells
Human hepatocytes	$t_{1/2}$ = 139 min<br>CL_{int} = 10.0 µL/min/10^6 cells
Kinetic solubility (pH 2.0 / 6.5)	>320 / 160-320 µM
gLogD (pH 7.4)	3.8
m/h PPB	93.8 / 84.7 %
hERG binding	IC_{50} = 5.39 µM
CYP450 (7 isoforms)	IC_{50} = >20 µM (1A2, 2B6, 2C8, 2C9 2C19, 3A4/5), 13.8 µM (2D6)
CYP3A4/5 (time dependent)	IC_{50} = >20 µM (-NADPH)<br>>20 µM (+NADPH)

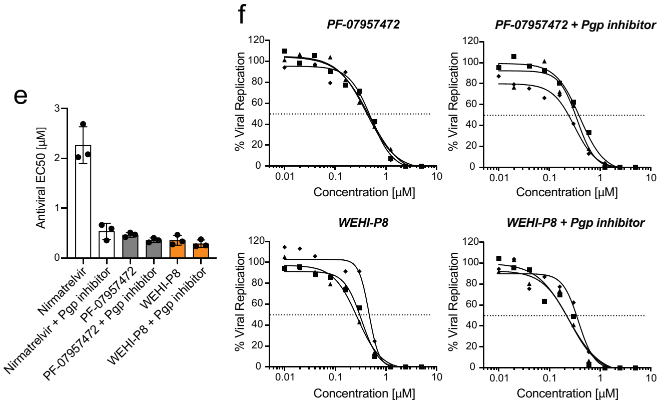

Supplementary Figure 6. Properties of *WEHI-P8.*

(a) Biochemical IC_{50} of ***WEHI-P8*** is displayed as the mean of n = 2 independent experiments each with technical duplicates. A representative curve is shown. See **Methods** for further assay details **(b)** SPR binding assay (K_D) of ***WEHI-P8.*** Values for SPR indicate the mean of n = 2 experiments and a representative graph is shown. **(c)** Cellular FRET assay (cellular EC_{50}) of ***WEHI-P8***. Data is displayed as the mean from two independent experiments (n=2) each with technical triplicates. **(d)** DMPK properties for ***WEHI-P8***. CL_{int} , Intrinsic clearance; gLogD, partition co-efficient estimation using a gradient chromatography method; m/h PPB, mouse/human protein plasma binding; hERG, human ether-à-go-go-related gene; CYP450, cytochrome P450. ***WEHI-P8*** showed moderate stability in mouse microsomes ($T_{1/2}$ = 41.5 min, CL_{int} = 33.4 µL/min/mg) but low clearance in human microsomes ($T_{1/2}$ = 144 min, CL_{int} = 9.7 µL/min/mg). This trend continued with modest stability in mouse ($T_{1/2}$ = 82.6 min, CL_{int} = 16.8 µL/min/10^6 cells) and greater stability in human ($T_{1/2}$ = 139 min, CL_{int} = 10.0 µL/min/10^6 cells) hepatocytes. Protein plasma binding in mouse and human was 93.8% and 84.7%, respectively. Off-target effects of ***WEHI-P8*** include low hERG binding (measured by fluorescence polarisation) (IC_{50} = 5.39 µM), high selectivity against 7 CYPs (IC_{50} = >20 µM, 1A2, 2B6, 2C8, 2C9, 2C19, 3A4/5; 13.8 µM 2D6) and no time-dependent inhibition of CYP3A4/5 with or without NADPH (IC_{50} = >20 µM). **(e)** Antiviral plaque assay to assess activity of ***WEHI-P8*** on viral replication. Nirmatrelvir (see **Supplementary Figure 2e**) and PF-07957472[3] were used to benchmark ***WEHI-P8*** with or without the Pgp inhibitor CP100356 (2 µM). Data is displayed as the mean of

experiments performed in triplicate. **(f)** Corresponding viral inhibition curves for data shown in **(e)** (for nirmatrelvir see **Supplementary Figure 2e**).

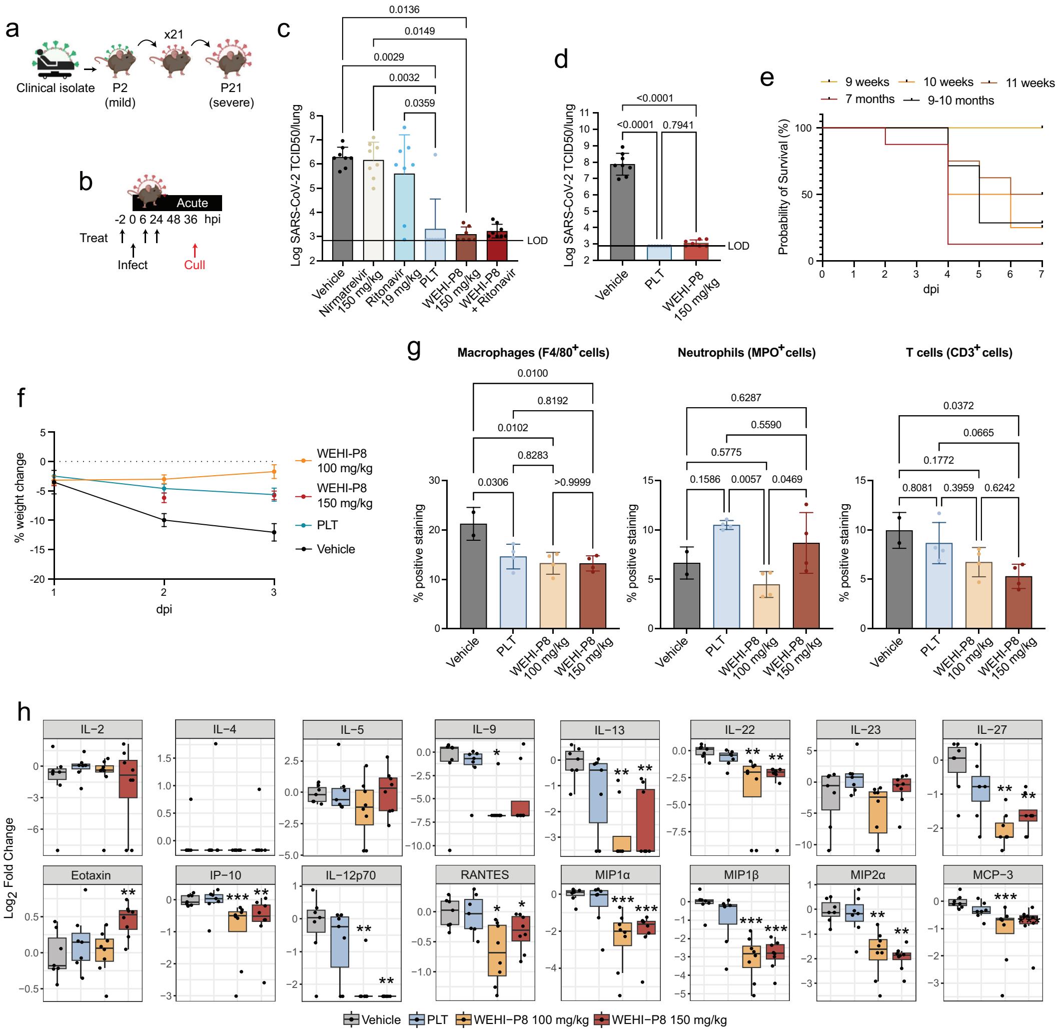
a
Clinical isolate
P2 (mild)
x21
P21 (severe)
b
Acute
-2 0 6 24 48 36 hpi
Treat
Infect
Cull
c
Log SARS-CoV-2 TCID50/lung
0.0136
0.0149
0.0029
0.0032
0.0359
LOD
Vehicle
Nirmatrelvir 150 mg/kg
Ritonavir 19 mg/kg
PLT
WEHI-P8 150 mg/kg
WEHI-P8 + Ritonavir
d
<0.0001
<0.0001
0.7941
LOD
Vehicle
PLT
WEHI-P8 150 mg/kg
e
9 weeks
10 weeks
11 weeks
7 months
9-10 months
Probability of Survival (%)
dpi
f
% weight change
dpi
WEHI-P8 100 mg/kg
WEHI-P8 150 mg/kg
PLT
Vehicle
g
Macrophages (F4/80+cells)
Neutrophils (MPO+cells)
T cells (CD3+cells)
% positive staining
0.0100
0.8192
0.0102
0.8283
0.0306
>0.9999
0.6287
0.5590
0.5775
0.1586
0.0057
0.0469
0.0372
0.0665
0.1772
0.8081
0.3959
0.6242
Vehicle
PLT
WEHI-P8 100 mg/kg
WEHI-P8 150 mg/kg
h
Log2 Fold Change
IL-2
IL-4
IL-5
IL-9
IL-13
IL-22
IL-23
IL-27
Eotaxin
IP-10
IL-12p70
RANTES
MIP1α
MIP1β
MIP2α
MCP-3
Vehicle
PLT
WEHI-P8 100 mg/kg
WEHI-P8 150 mg/kg

Supplementary Figure 7. *WEHI-P8* is effective in acute models of mild and severe SARS-CoV-2 infection.

(a) Schematic of the serial passage method used to generate the SARS-CoV-2 strains causing mild (P2) and severe (P21) disease[4]. **(b)** Schematic showing treatment regime used in **c and Supplementary Figure 8**. **(c)** C57BL/6 (WT) mice were infected with P2 and treated with either vehicle, nirmatrelvir (150 mg/kg) or ritonavir (19 mg/kg) alone, PLT (56 mg/kg nirmatrelvir, 19 mg/kg ritonavir), ***WEHI-P8*** (150 mg/kg), or the combination of WEHI-P8 and ritonavir. Mice were monitored for viral burden at 3 dpi (n_{veh}=8, n_{PLT}=8, n_{Nirma}=8, n_{Ritona}=8, n_{P8}=7, $n_{P8+ritona}$=8 mice per group, mean ± SD). **(d)** WT mice were infected with P2 and treated with either vehicle, PLT, or ***WEHI-P8*** (150 mg/kg) (see schematic **Fig. 3c**). Mice were monitored for viral burden at 3 dpi (n_{veh}=8, n_{PLT}=8, n_{P8}=7, mean ± SD) **(e)** WT mice different ages were infected with P21 and monitored to determine the proportion of mice that became severely ill, reaching predetermined ethical endpoint (n_{7m}=8, $n_{9\text{-}10m}$=7, n_{11w}=8, n_{9w}=6, n_{10w}=4 per group). **(f-h)** WT mice were infected with P21 and treated with vehicle, PLT (56 mg/kg nirmatrelvir, 19 mg/kg ritonavir), or ***WEHI-P8*** (150 mg/kg) (schematic **Fig. 3c**) **(f)** percent weight change compared to initial weight is shown **(g)** Representative images of haematoxylin and eosin (H&E) and immunohistochemistry (IHC) stained lungs probed for F4/80 (macrophages), myeloperoxidase (MPO, neutrophils) and CD3 (T cells) are shown. Images are representative of 2 (vehicle) and 4 (treated) animals per group. Two independent experiments were performed. Scale bar = 250 µm. **(h)** Levels of cytokines and chemokines measured by ELISA of lung homogenates from mice infected with P21. $n_{vehicle}$=7, n_{PLT}=7, $n_{P8\text{-}100}$=8, $n_{P8\text{-}150}$=8 mice per group; boxplots depict the median and

interquartile range (IQR). Whiskers extend to the furthest data point within 1.5 times the IQR from each box end. P-values are shown above each group and were determined by **(c,d)** one-way ANOVA with Tukey's multiple comparisons tests after $\log_{10}$ transformation **(g)** one-way ANOVA with Tukey's multiple comparisons tests and **(h)** Wilcoxon rank-sum test, with Bonferroni adjustment for multiple comparisons. **Source data** are available. **Supplementary Fig. 7a-b** Partially created in BioRender. M. Bader, S. (2025) https://BioRender.com/i43m477

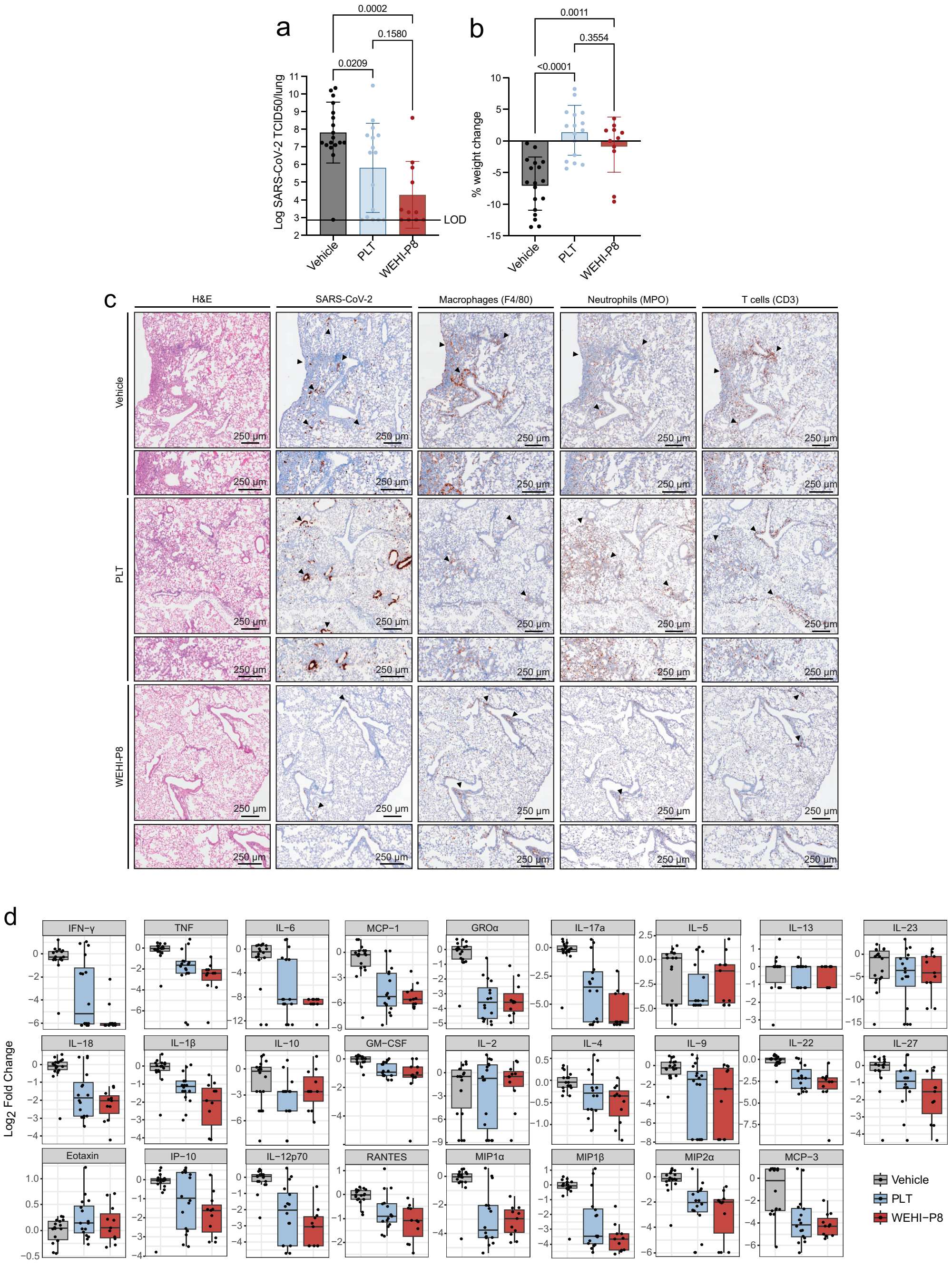
a
0.0002
0.1580
0.0209
Log SARS-CoV-2 TCID50/lung
LOD
Vehicle
PLT
WEHI-P8
b
0.0011
0.3554
<0.0001
% weight change
Vehicle
PLT
WEHI-P8
c
H&E
SARS-CoV-2
Macrophages (F4/80)
Neutrophils (MPO)
T cells (CD3)
Vehicle
PLT
WEHI-P8
250 µm
d
IFN−γ
TNF
IL−6
MCP−1
GROα
IL−17a
IL−5
IL−13
IL−23
IL−18
IL−1β
IL−10
GM−CSF
IL−2
IL−4
IL−9
IL−22
IL−27
Eotaxin
IP−10
IL−12p70
RANTES
MIP1α
MIP1β
MIP2α
MCP−3
Log2 Fold Change
Vehicle
PLT
WEHI−P8

Supplementary Figure 8. WEHI-P8 administered pre-infection is effective in acute models of severe SARS-CoV-2 infection.

(a-d) WT mice were infected with the P21 strain of SARS-CoV-2 (see **Supplementary Fig. 7a**) and treated with either vehicle, PLT, or ***WEHI-P8*** (150 mg/kg) at -2h, 6h and 24h (see schematic **Supplementary Figure 7b** and **Methods**). At 3 days post-infection (dpi), mice were monitored for **(a)** viral burden and **(b)** percent weight change compared to initial weight. Results are pooled from 2 independent experiments, n_{veh}=18, n_{PLT}=16, n_{P8}=11 mice per group, mean ± SD). **(c)** Histological analysis of fixed lungs. Haematoxylin and eosin (H&E) and immunohistochemistry (IHC) stained lungs are shown. Markers used for each cell type are indicated in brackets and images are representative from 4 animals per condition. Scale bars = 250 µm. **(d)** Levels of cytokines and chemokines measured by ELISA of lung homogenates from mice infected with SARS-CoV-2 P21 strain (n_{veh}=18, n_{PLT}=16, n_{P8}=11 mice per group; boxplots depict the median and interquartile range (IQR). Whiskers extend to the furthest data point within 1.5 times the IQR from each box end. P-values are shown above each group and were determined by **(a)** one-way ANOVA with Tukey's multiple comparisons tests after $\log_{10}$ transformation, **(b)** one-way ANOVA with Tukey's multiple comparisons tests and **(d)** Wilcoxon rank-sum test, with Bonferroni adjustment for multiple comparisons. **Source data** are available.

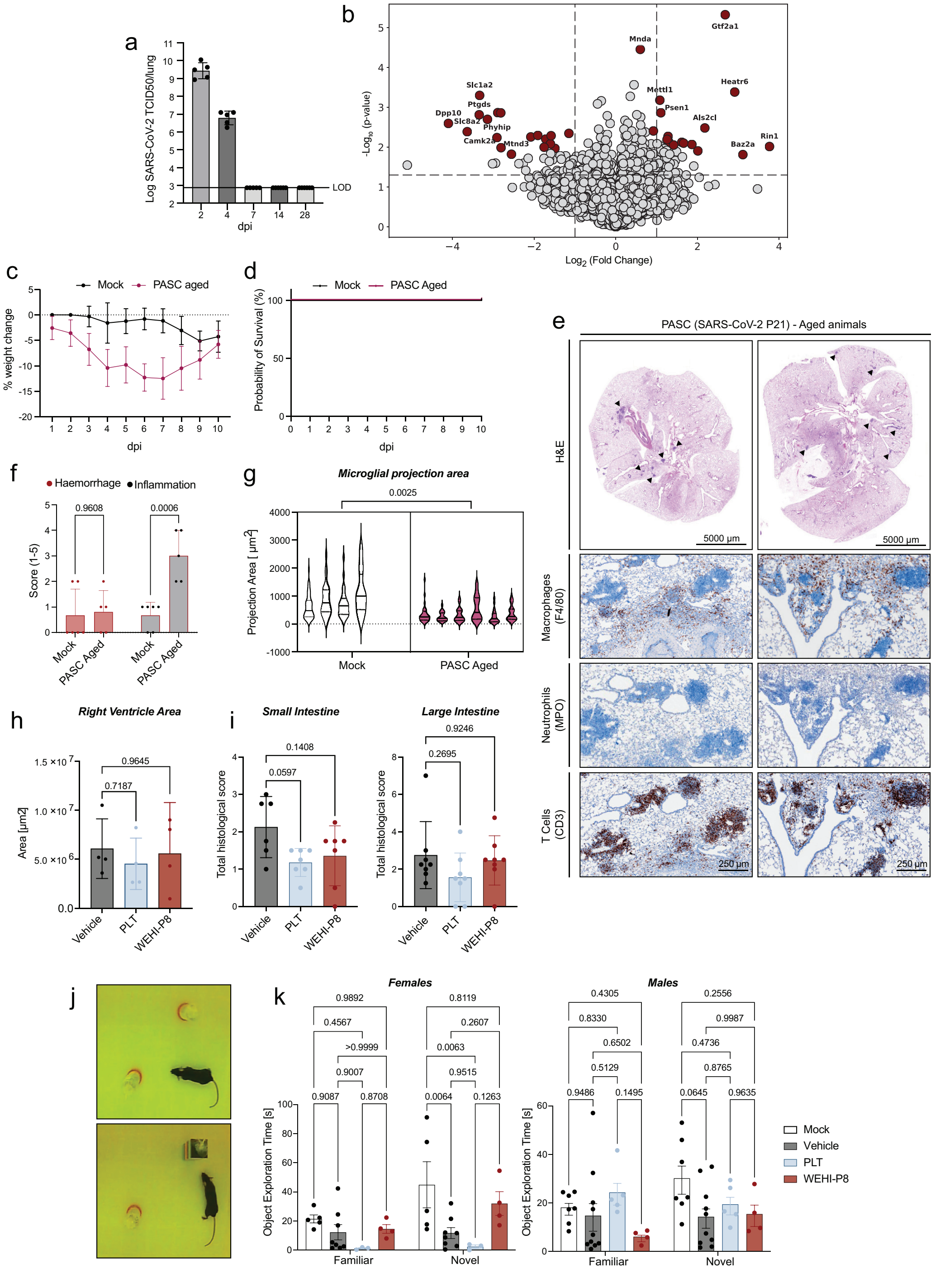
a
Log SARS-CoV-2 TCID50/lung
LOD
dpi
b
Gtf2a1
Mnda
Heatr6
Slc1a2
Mettl1
Ptgds
Psen1
Dpp10
Slc8a2
Als2cl
Phyhip
Rin1
Camk2a
Mtnd3
Baz2a
$-Log_{10}$ (p-value)
Log_2 (Fold Change)
c
Mock
PASC aged
% weight change
dpi
d
Mock
PASC Aged
Probability of Survival (%)
dpi
e
PASC (SARS-CoV-2 P21) - Aged animals
H&E
5000 µm
Macrophages (F4/80)
Neutrophils (MPO)
T Cells (CD3)
250 µm
f
Haemorrhage
Inflammation
Score (1-5)
0.9608
0.0006
Mock
PASC Aged
g
Microglial projection area
0.0025
Projection Area [µm²]
Mock
PASC Aged
h
Right Ventricle Area
Area [µm2]
0.9645
0.7187
Vehicle
PLT
WEHI-P8
i
Small Intestine
Large Intestine
Total histological score
0.1408
0.0597
0.9246
0.2695
j
k
Females
Males
Object Exploration Time [s]
0.9892
0.4567
>0.9999
0.9007
0.9087
0.8708
0.8119
0.2607
0.0063
0.9515
0.0064
0.1263
0.4305
0.8330
0.6502
0.5129
0.9486
0.1495
0.2556
0.9987
0.4736
0.8765
0.0645
0.9635
Familiar
Novel
Mock
Vehicle
PLT
WEHI-P8

Supplementary Figure 9. A multi-organ PASC model to test antiviral efficacy.

(a) C57BL/6 (WT) mice were infected with SARS-CoV-2 P21 and monitored at different days post-infection (dpi) for lung viral burden (n_{28dpi}=6, n_{6dpi}=14, n_{2dpi}=5, n_{7dpi}=5, n_{4dpi}=5 mice per group; mean ± SD). **(b)** Mice were challenged with either mock or P21 and lungs were taken at 45 dpi for proteomics analysis. Volcano plot of proteins regulated at 45 dpi versus mock is shown. n_{mock}=4, n_{pasc}=5. **(c-g)** WT aged mice (>6 months) were infected with 200 TCID50 of P21 (see **Fig. 4a**) and monitored daily during acute infection for **(c)** percent weight change and **(d)** percent of animals requiring euthanasia (n_{mock}=6, n_{pasc}=8, results are representative of 2 independent experiments). **(e)** lungs of aged, recovered animals at 3 months post-infection (mpi) were analysed via haematoxylin and eosin (H&E) and immunohistochemistry (IHC). Images are representative of 5 animals. Scale bars = 5000 or 250 µm. **(f)** H&E-stained lungs were assessed for inflammatory foci and haemorrhage, with pathology scored on a scale from 0 to 5 (Methods) (n_{mock}=6, n_{pasc}=5). **(g)** Quantification of the projection area of IBA-1 positive cells in the hippocampus (n_{mock}=4, n_{pasc}=6 animals per group, >20 cells per mouse counted; violin plots show median and quartiles). **(h-k)** Mice were treated as described in **Supplementary Fig. 8a. (h)** Hearts were collected for H&E staining and right ventricle area was quantified (n=4 mice per group; mean ± SD). **(i)** Histological analysis of H&E-stained intestines at 1 mpi. Total histological score of the small (n = n_{veh}=6, n_{P8}=7, n_{PLT}=7 animals per group) and large intestines (n=8 animals per group) is shown (Mean ± SD). **(j)** Exemplary images of novel object recognition test. Top: animals are introduced to a box containing two objects of the same kind. Bottom: after 1 h break, animals are introduced to the same box, which now contains a novel object. Time spent

exploring the objects is quantified and shown in **(k)**. P-values are shown on the respective graphs and were determined by **(f,k)** Two-way ANOVA with Sidak's multiple comparisons tests **(h,i)** One-way ANOVA with Tukey's multiple comparisons tests (**e,g**) and (**g**) nested two-tailed t-test. **Source data** is available.

	SARS-CoV-2 PLpro bound to ***WEHI-P1***	SARS-CoV-2 PLpro bound to ***WEHI-P2***	SARS-CoV-2 PLpro bound to ***WEHI-P4***	SARS-CoV-2 PLpro bound to ***WEHI-P24***
PDB ID	9CYB	9CYC	9CYD	9CYK
**Data collection**				
Space group	$P\,2\,2_1\,2_1$	$P\,2\,2_1\,2_1$	$P\,4_3\,3\,2$	$P\,2\,2_1\,2_1$
Cell dimensions				
a, *b*, *c* (Å)	66.46, 71.07, 176.12	66.58, 71.30, 177.51	156.59, 156.59, 156.59	67.12, 71.45, 176.45
α, β, γ (°)	90.00, 90.00, 90.00	90.00, 90.00, 90.00	90.00, 90.00, 90.00	90.00, 90.00, 90.00
Resolution (Å)	48.54 – 1.98 (2.03 – 1.98)	48.66 – 2.01 (2.06 – 2.01)	49.52 – 2.80 (2.95 – 2.80)	48.92 – 1.88 (1.92 – 1.88)
R_{merge} (within I+/I-)	0.119 (1.441)	0.066 (1.041)	0.295 (5.095)	0.062 (1.479)
Rpim (within I+/I-)	0.048 (0.580)	0.050 (0.806)	0.065 (1.129)	0.026 (0.610)
Half-Set Correlation (CC1/2)	0.999 (0.835)	0.993 (0.680)	0.999 (0.585)	1.000 (0.642)
$I/\sigma I$	13.4 (1.9)	9.4 (1.1)	12.9 (1.0)	12.1 (1.1)
Completeness (%)	99.9 (98.2)	99.6 (95.7)	100 (100)	99.6 (94.9)
Redundancy	13.6 (13.6)	4.6 (4.6)	39.8 (39.9)	6.7 (6.6)
**Refinement**				
Resolution (Å)	37.43 – 1.98	46.93 – 2.01	49.52 – 2.80	48.92 – 1.88
No. reflections	58896	57062	16726	69303
R_{work} / R_{free}	0.181/ 0.208	0.206/ 0.237	0.241/ 0.265	0.181/ 0.212
No. atoms				
Protein	4744	4732	2450	4744
Ligand/ion	66	86	32	74
Water	253	188	0	344
B-factors				
Protein	45.1	59.0	98	48.8
Ligand/ion	36.8	61.5	69.1	42.9
Water	42.4	52.8	-	49.1
R.m.s. deviations				
Bond lengths (Å)	0.007	0.003	0.008	0.005
Bond angles (°)	0.823	0.624	0.955	0.827

*All datasets were collected from a single crystal each. *Values in parentheses are for highest-resolution shell.

Supplementary Table 1. Data collection and refinement statistics of WEHI-P bound structures. Numbers in brackets correspond to the highest resolution shell.

Uncropped Gel

Uncropped gel from Supplementary Fig. 4c.

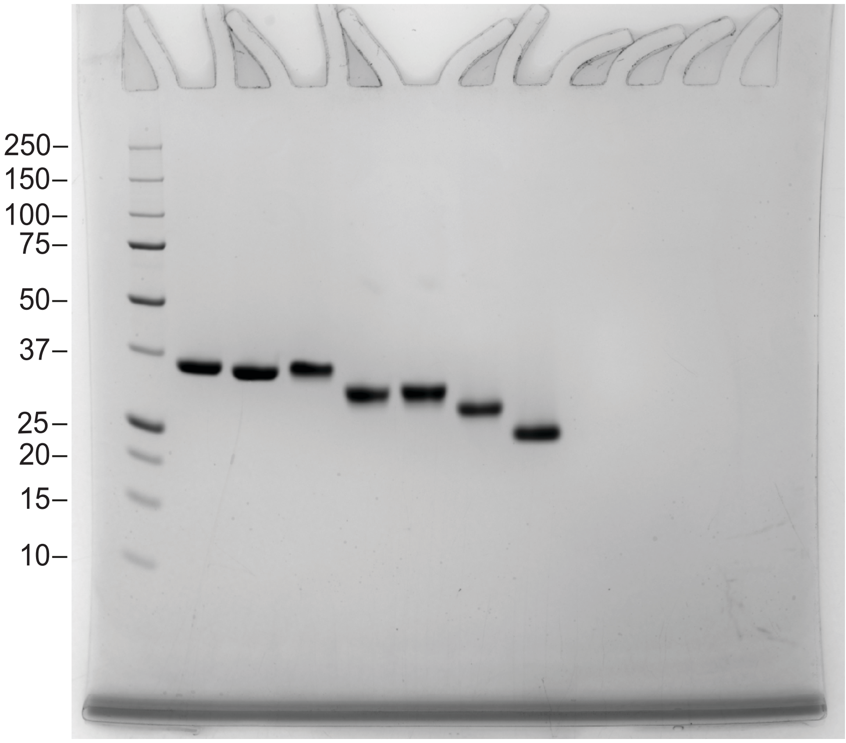

Medicinal Chemistry – General Experimental

All reagents were used as received from commercial suppliers unless otherwise stated. NMR spectra were recorded at ambient temperature on a Bruker Avance 300 MHz instrument in the specified deuterated solvent. Observed proton chemical shifts were reported as units of parts per million (ppm) relative to the respective residual solvent peak DMSO-d6 (δ 2.50). Multiplicities were reported: s (singlet), d (doublet), t (triplet), q (quartet), dd (doublet of doublets), dt (doublet of triplets) and m (multiplet). Coupling constants were reported as a *J* value in Hertz (Hz). HRMS were acquired through a Thermo Scientific™ nano-LC Q Exactive™ Plus Mass spectrometer or an Agilent 6530 TOF LC/MS Mass Spectrometer coupled to an Agilent 1290 Infinity (Agilent, Palo Alto, CA). All data were acquired, and reference mass corrected via a dual electrospray ionisation (ESI) source. Acquisition was performed using the Agilent OpenLab ECM XT software. Abbreviations: DCM (dichloromethane), DMF (*N*,*N*-dimethylformamide), EtOH (ethanol), EtOAc (ethyl acetate), FA (formic acid), MeCN (acetonitrile), MeOH (methanol), THF (tetrahydrofuran), TFA (trifluoracetic acid).

General HPLC Methods

Achiral purity for all compounds were acquired on a Waters Acquity UPLC with an Acquity BEH C-18 (2.1 x 100mm, 1.7 μm) column using the following step-wise gradient method at 0.3 mL/min;

Time (min)	A: 5mM $NH_4CH_3CO_2$ in H_2O	B: MeCN
0.0	90.0	10.0

1.0	90.0	10.0
2.0	85.0	15.0
4.5	45.0	55.0
6.0	10.0	90.0
8.0	10.0	90.0

With the exception of (***WEHI-P70***) which was acquired using the following step-wise gradient at 0.3 mL/min;

Time (min)	A: 5mM $NH_4CH_3CO_2$ in H_2O	B: MeCN
0.0	90.0	10.0
1.0	90.0	10.0
2.0	85.0	15.0
4.5	70.0	30.0
6.0	55.0	45.0
8.0	20.0	80.0
10.0	10.0	90.0

Chiral ee for all compounds was acquired on a Waters Acquity UPC2 with a Chiralcel OX-H (4.6 x 250mm), 5μM with CO_2/0.2% NH_4OH in MeOH, (70:30) (v/v) at 3 mL/min with the exception of (***WEHI-P70***) which was acquired on a Waters Acquity UPLC using

a Chiralcel OJH (4.6 x 250mm), 5μM with 0.1% NH_4OH in MeOH, (100%) (v/v) at 1 mL/min. All HPLC data processing performed using Waters Empower Software.

Synthetic procedures

Synthesis of (1-cyclopentylpiperidin-3-yl)(6-methoxynaphthalen-2-yl)methanone (***WEHI-P1***) and (*R*,*E*)-(1-cyclopentylpiperidin-3-yl)(6-methoxynaphthalen-2-yl)methanone *O*-methyl oxime (***WEHI-P2***) **(Supplementary Figure 10).**

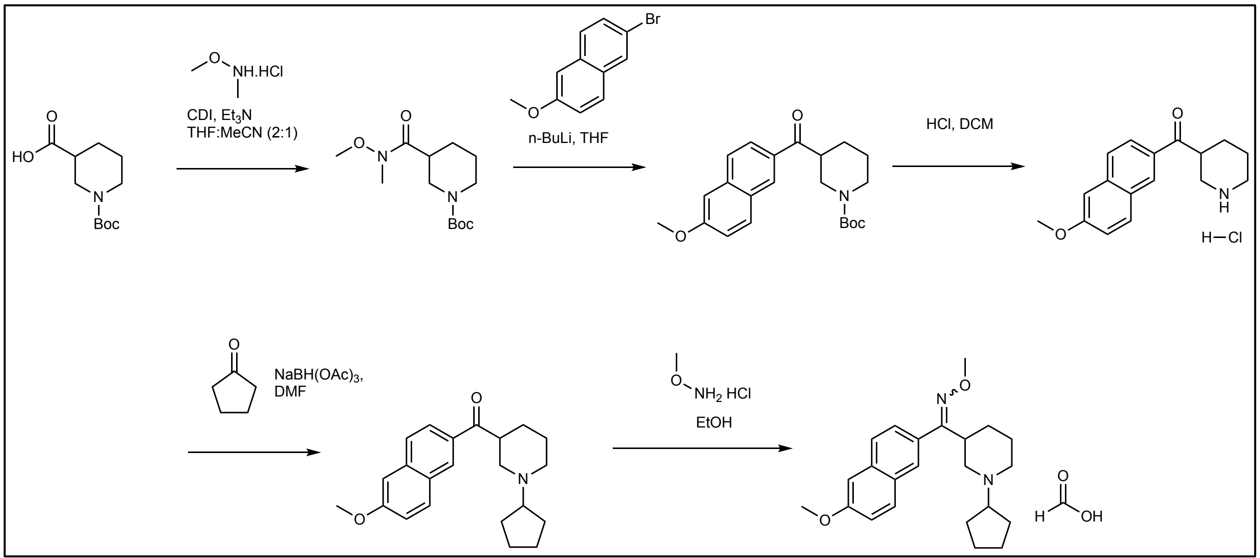

Supplementary Figure 10: Synthesis of **WEHI-P1** and **WEHI-P2**

tert-Butyl 3-(methoxy(methyl)carbamoyl)piperidine-1-carboxylate

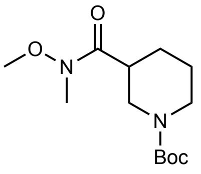

To a stirred solution of *tert*-butoxycarbonylpiperidine-3-carboxylic acid (25.0 g, 109 mmol) in THF (200 mL) at 0°C was added 1,1-carbonyldimidazole (21.2 g, 1.2 eq., 131 mmol). The resulting mixture was stirred for 1h. Separately, methoxy(methyl)amine hydrochloride (16.7 g, 2.5 eq., 273 mmol) and Et_3N (25.2 mL, 1.6 eq., 174 mmol) were combined in MeCN (100 mL) at 0 °C and stirred for 1h. The two solutions were then combined and stirred at ambient temperature for 16h. The reaction mixture was quenched with water and extracted with DCM (3 x 200 mL). The combined organic layers were washed with sat. NaCl, dried

over Na_2SO_4, evaporated under reduced pressure, and purified by SiO_2 column chromatography (5% MeOH in DCM) to afford *tert*-butyl 3-(methoxy(methyl)carbamoyl)piperidine-1-carboxylate (27.5 g, 91%) as a white solid.

tert-Butyl 3-(6-methoxy-2-naphthoyl)piperidine-1-carboxylate

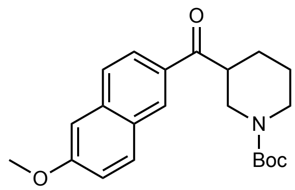

To a stirred solution of 2-bromo-6-methoxynaphthalene (20.0 g, 84.4 mmol) in THF (200 mL) cooled at -78°C was added 2.5M *n*-BuLi in hexane (67.5 mL, 2.0 eq., 169 mmol) dropwise under nitrogen atmosphere. The reaction mixture was stirred for 1h at -78°C and then a solution of *tert*-butyl 3-(methoxy(methyl)carbamoyl)piperidine-1-carboxylate (27.6 g, 1.2 eq., 101 mmol) in tetrahydrofuran (67 mL) was then added dropwise and the reaction mixture stirred overnight at ambient temperature. The reaction mixture was quenched with sat. NH_4Cl solution and extracted with EtOAc (3 x 300 mL). The combined organics were washed with sat. NaCl, dried over Na_2SO_4, evaporated under reduced pressure and purified by SiO_2 column chromatography (20% EtOAc/n-heptane) to afford *tert*-butyl 3-(6-methoxy-2-naphthoyl)piperidine-1-carboxylate (25.0 g, 64%).

(6-Methoxynaphthalen-2-yl)(piperidin-3-yl)methanone (HCl salt)

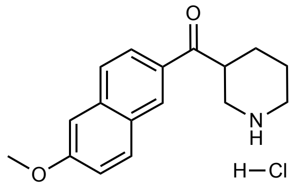

To a stirred solution of *tert*-butyl 3-(6-methoxy-2-naphthoyl)piperidine-1-carboxylate (8.00 g, 21.7 mmol) in DCM (40 mL) at 0°C was added 4M hydrogen chloride in dioxane (54.1 mL, 10 eq., 217 mmol) dropwise under nitrogen atmosphere. The reaction mixture was stirred for 3h. The reaction mixture was concentrated to dryness and the crude material

triturated with MeCN to afford (6-methoxynaphthalen-2-yl)(piperidin-3-yl)methanone as a hydrochloride salt (5.70 g, 85%) as a white solid.

(1-Cyclopentylpiperidin-3-yl)(6-methoxynaphthalen-2-yl)methanone **(*WEHI-P1*)**

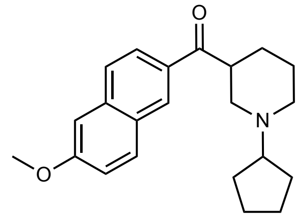

To a stirred solution of (6-methoxynaphthalen-2-yl)(piperidin-3-yl)methanone (HCl salt) (1.00 g, 3.27 mmol) and cyclopentanone (868 mL, 3 eq., 9.81 mmol) in DMF (10 mL) under nitrogen atmosphere was added $NaBH(OAc)_3$ (1.04 g, 1.5 eq., 4.91 mmol). The reaction mixture was stirred at ambient temperature for 12h. The reaction mixture was poured on ice-cold water and extracted with EtOAc (3 x 50 mL). The combined organics were washed with sat. NaCl solution, dried over Na_2SO_4, filtered, and evaporated under reduced pressure and purified by SiO_2 column chromatography (5% MeOH in DCM) to afford (1-cyclopentylpiperidin-3-yl)(6-methoxynaphthalen-2-yl)methanone **(*WEHI-P1*)** (600 mg, 53%, >98% purity) as a pale yellow oil. ^{1}H NMR (300 MHz, DMSO) δ 8.61 (s, 1H), 8.06 (d, J = 9.0 Hz, 1H), 7.95 – 7.86 (m, 2H), 7.40 (d, J = 2.5 Hz, 1H), 7.25 (dd, J = 9.0, 2.5 Hz, 1H), 3.91 (s, 3H), 3.72 (tt, J = 10.7, 3.5 Hz, 1H), 3.04 (d, J = 11.2 Hz, 1H), 2.93 (d, J = 11.1 Hz, 1H), 2.50 (m, 1H), 2.08 (t, J = 10.8 Hz, 1H), 1.98 – 1.20 (m, 13H). ^{13}C NMR (75 MHz, DMSO) δ 201.6 (C), 159.4 (C), 136.9 (C), 131.4 (CH), 131.0 (C), 129.8 (CH), 127.6 (C), 127.2 (CH), 124.5 (CH), 119.5 (CH), 106.0 (CH), 66.7 (CH), 55.4 (CH_3), 54.8 (CH_2), 52.0 (CH_2), 43.4 (CH), 29.8 (CH_2), 29.7 (CH_2), 27.9 (CH_2), 24.4 (CH_2), 23.8 (CH_2), 23.7 (CH_2). HR-ESMS calcd. for $C_{22}H_{28}NO_2^+$ [M + H] 338.2115, found 338.2129.

(1-Cyclopentylpiperidin-3-yl)(6-methoxynaphthalen-2-yl)methanone *O*-methyl oxime (formic acid salt) ***(WEHI-P2)***

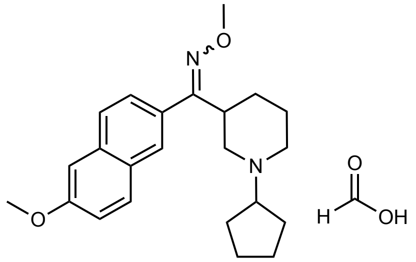

To a stirred solution of (1-cyclopentylpiperidin-3-yl)(6-methoxynaphthalen-2-yl)methanone (120 mg, 0.356 mmol) in EtOH (16 mL) under nitrogen atmosphere was added *O*-methylhydroxylamine hydrochloride (89.1 mg, 3 eq., 1.07 mmol). The reaction mixture was stirred at 90°C for 12h. The reaction mixture was evaporated under reduced pressure and poured into ice cold water and extracted with EtOAc (3 x 80 mL). The combined organic layers were washed with ice cold water, sat. NaCl solution, dried over Na_2SO_4, filtered, and evaporated under reduced pressure and purified using XSelect C18(19x250)mm.10μ, 0.1% FA in H_2O/MeCN to afford (1-cyclopentylpiperidin-3-yl)(6-methoxynaphthalen-2-yl)methanone *O*-methyl oxime as a formic acid salt ***(WEHI-P2)*** (60 mg, 46%, >99% purity). As determined by ^{1}H NMR the relative ratio of E/Z isomers occur in a ratio of 0.66 (Z) to 0.33 (E). ^{1}H NMR (300 MHz, DMSO) δ (major isomer): 7.93 – 7.74 (m, 3H), 7.39 – 7.31 (m, 2H), 7.20 – 7.16 (m, 1H), 3.88 (s, 3H), 3.71 (s, 3H), 3.06 – 2.75 (m, 4H), 2.08 – 1.86 (m, 2H), 1.83 – 1.20 (m, 12H); (minor isomer): 8.26 (s, 1H), 7.93 – 7.74 (m, 3H), 7.52 (dd, J = 8.5, 1.7 Hz, 1H), 7.39 – 7.31 (m, 1H), 7.20 – 7.16 (m, 1H), 3.88 (s, 6H), 3.49 – 3.36 (m, 1H), 3.06 – 2.75 (m, 2H), 2.60 – 2.51 (m, 1H), 2.26 (t, J = 11.1 Hz, 1H), 1.83 – 1.20 (m, 13H). ^{13}C NMR (75 MHz, DMSO) δ 160.3, 159.2, 157.8, 134.1, 134.0, 130.5, 129.8, 129.7, 129.0, 127.9, 127.8, 126.7, 126.5, 126.3, 126.2, 126.1, 125.8, 119.4, 118.9, 105.8, 105.8, 66.7, 61.6, 61.2, 55.6, 55.3, 53.7, 51.8, 41.8, 37.5, 29.7, 29.6, 29.4, 28.5, 26.7, 25.0, 24.5, 23.6, 23.5. HR-ESMS calcd. for $C_{23}H_{31}N_2O_2^+$ [M + H] 367.2380, found 367.2397.

Synthesis of (*E*)-((*R*)-1-((1s,4S)-4-hydroxycyclohexyl)piperidin-3-yl)(6-methoxynaphthalen-2-yl)methanone *O*-methyl oxime **(*WEHI-P4*) (Supplementary Figure 11).**

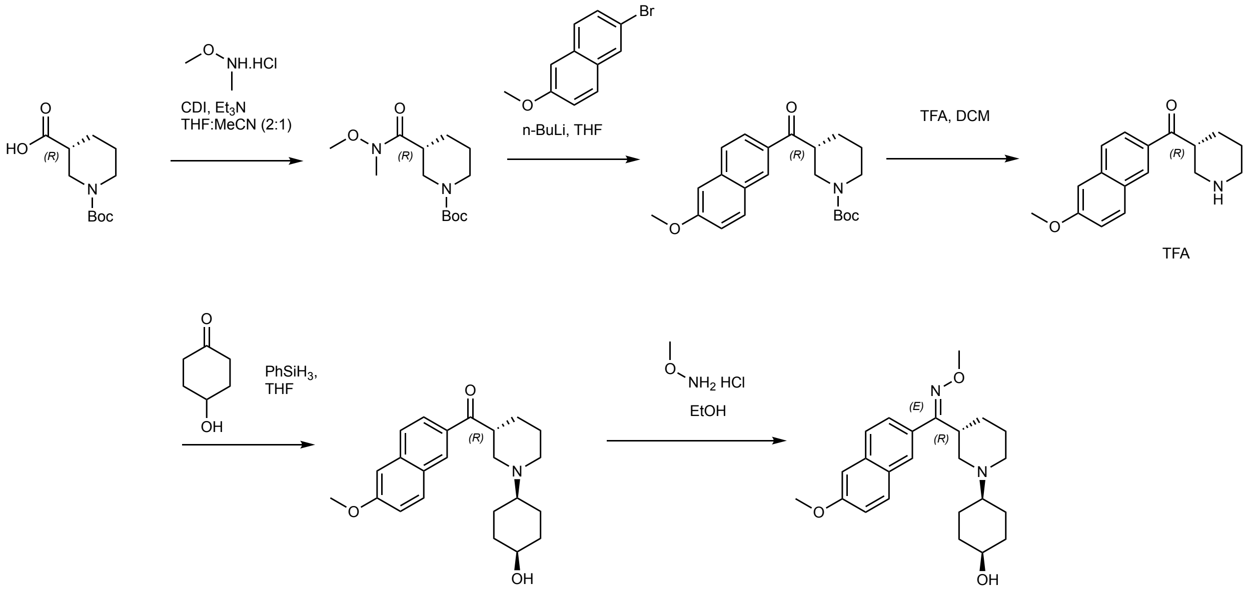

Supplementary Figure 11: Synthesis of **WEHI-P4**

(*R*)-*tert*-butyl 3-(methoxy(methyl)carbamoyl)piperidine-1-carboxylate

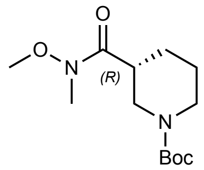

To a stirred solution of (*R*)-1-*tert*-butoxycarbonyl)piperidine-3-carboxylic acid (15.00 g, 65.4 mmol) in THF (100 mL) at 0°C was added 1,1-carbonyldimidazole (12.73 g, 1.2 eq., 78.5 mmol). The resulting mixture was stirred for 1h. Separately, methoxy(methyl)amine hydrochloride (15.95 g, 2.5 eq., 163.5 mmol) and Et_3N (14.58 mL, 1.6 eq., 105 mmol) were combined in MeCN (100 mL) at 0°C and stirred for 1h. The two solutions were then combined and stirred at ambient temperature for 16h. The reaction mixture was quenched with water and extracted with DCM (3 x 200 mL). The combined organic layers were washed with sat. NaCl, dried over Na_2SO_4, evaporated under reduced pressure, and purified by SiO_2 column

chromatography (5% MeOH in DCM) to afford (*R*)-*tert*-butyl 3-(methoxy(methyl)carbamoyl)piperidine-1-carboxylate (15.02 g, 84%) as a semi-solid.

(*R*)-*tert*-butyl 3-(6-methoxy-2-naphthoyl)piperidine-1-carboxylate

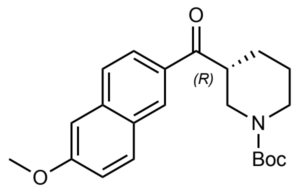

To a stirred solution of 2-bromo-6-methoxynaphthalene (20.0 g, 84.4 mmol) in THF (200 mL) cooled at -78°C was added 2.5M n-BuLi in hexane (67.5 mL, 2.0 eq., 169 mmol) dropwise under nitrogen atmosphere. The reaction mixture was stirred for 1h at -78°C and then a solution of (*R*)-*tert*-butyl 3-(methoxy(methyl)carbamoyl)piperidine-1-carboxylate (27.6 g, 1.2 eq., 101 mmol) in tetrahydrofuran (67 mL) was added dropwise and the reaction mixture stirred overnight at ambient temperature. The reaction mixture was quenched with sat. NH_4Cl solution and extracted with EtOAc (3 x 300 mL). The combined organic layer was washed with sat. NaCl, dried over Na_2SO_4, evaporated under reduced pressure and purified through SiO_2 column chromatography (20% EtOAc in n-heptane) to afford (*R*)-*tert*-butyl 3-(6-methoxy-2-naphthoyl)piperidine-1-carboxylate (31.2 g, 99%).

(*R*)-(6-Methoxynaphthalen-2-yl)(piperidin-3-yl)methanone (TFA salt)

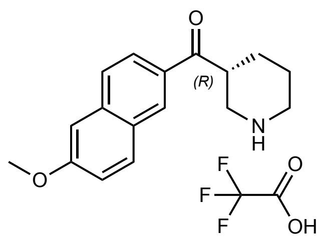

To a stirred solution of *tert*-butyl (3R)-3-(6-methoxynaphthalene-2-carbonyl)piperidine-1-carboxylate (31.0 g, 83.9 mmol) in DCM (93 mL) was added TFA (19.3 mL, 3 eq., 252 mmol) slowly at 0°C under nitrogen atmosphere. The reaction mixture was stirred at ambient temperature for 6h, evaporated under reduced

pressure and triturated with *n*-pentane to afford (*R*)-(6-methoxynaphthalen-2-yl)(piperidin-3-yl)methanone as a TFA salt (28.0 g, 91%) as a gum.

((R)-1-((1s*,4S*)-4-hydroxycyclohexyl)piperidin-3-yl)(6-methoxynaphthalen-2-yl)methanone

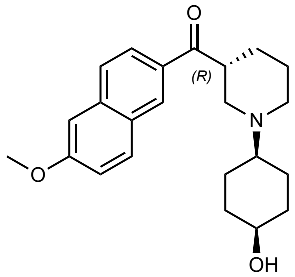

To a stirred solution of (*R*)-(6-methoxynaphthalen-2-yl)(piperidin-3-yl)methanone (TFA salt) (14.3 g, 39.1 mmol) in THF (180 mL) under nitrogen atmosphere was added 4-hydroxycyclohexanone (12.2 mL, 3 eq., 117.3 mmol) and the reaction mixture was stirred at ambient temperature for 1h. $PhSiH_3$ (14.5 mL, 3 eq., 117.3 mmol) was added and the reaction mixture was stirred at ambient temperature for 12h. The reaction mixture was poured onto ice-cold water and extracted with EtOAc (3 x 100 mL). The combined organic layers were washed with sat. NaCl solution, dried over Na_2SO_4, filtered, and evaporated under reduced pressure to give the crude compound. This was purified by RP-HPLC using Waters X-Bridge C18, 19x250mm, 10µ, 0.1% NH_4OH in H_2O/MeCN to obtain Peak 1 ((*R*)-1-((1s*,4S*)-4-hydroxycyclohexyl)piperidin-3-yl)(6-methoxynaphthalen-2-yl)methanone (5.15 g, 36%) and Peak 2 ((*R*)-1-((1r*,4R*)-4-hydroxycyclohexyl)piperidin-3-yl)(6-methoxynaphthalen-2-yl)methanone (7.70 g).

(*E*)-((*R*)-1-((1s*,4S*)-4-hydroxycyclohexyl)piperidin-3-yl)(6-methoxynaphthalen-2-yl)methanone *O*-methyl oxime **(*WEHI-P4*)**

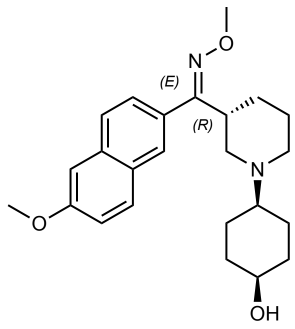

To a stirred solution of ((R)-1-((1s*,4S*)-4-hydroxycyclohexyl)piperidin-3-yl)(6-methoxynaphthalen-2-yl)methanone (5.8 g, 15.8 mmol) in EtOH (65 mL) under nitrogen atmosphere was added *O*-methylhydroxylamine hydrochloride (2.64 g, 2 eq., 31.6 mmol) and the reaction mixture was stirred at 90°C for 12h. The reaction mixture was evaporated under reduced pressure and poured into ice cold water and extracted with DCM (3 x 100 mL). The combined organic layers were washed with ice cold water, sat. NaCl solution, dried over Na_2SO_4, filtered, and evaporated under reduced pressure to give the crude compound. This was purified by RP-HPLC using Xtimate C4 (10x250mm) 10µ, 0.1% NH_4OH in H_2O/MeCN to obtain Peak 1 (1.7g) and Peak 2 (3.5 g). Peak 1 was further purified by chiral HPLC using Chiralcel OX-H, 30x250mm, 5µ, 0.1% NH_4OH in MeOH to afford Peak 1 (*E*)-((*R*)-1-((1s*,4S*)-4-hydroxycyclohexyl)piperidin-3-yl)(6-methoxynaphthalen-2-yl)methanone *O*-methyl oxime **(*WEHI-P4*)** (502 mg, 8%, >99% purity, >99% ee) as an off-white solid. ^{1}H NMR (300 MHz, DMSO) δ 7.90 (d, J = 9.0 Hz, 1H), 7.86 – 7.77 (m, 2H), 7.49 (dd, J = 8.5, 1.8 Hz, 1H), 7.33 (d, J = 2.6 Hz, 1H), 7.18 (dd, J = 8.9, 2.5 Hz, 1H), 4.23 (d, J = 3.4 Hz, 1H), 3.88 (s, 3H), 3.88 (s, 3H), 3.72 – 3.66 (m, 1H), 3.40 – 3.29 (m, 1H*), 2.88 – 2.75 (m, 2H), 2.37 (t, J = 10.8 Hz, 1H), 2.26 – 2.03 (m, 2H), 1.78 – 1.25 (m, 12H). *This signal was observed with the addition of D_2O. ^{13}C NMR (75 MHz, DMSO) δ 161.0 (C), 157.8 (C), 134.0 (C), 130.7 (C), 129.8 (CH), 127.8 (C), 126.7 (CH), 126.4 (CH), 125.9 (CH), 119.0 (CH), 105.8 (CH), 64.1 (CH), 62.3 (CH), 61.5 (CH_3), 55.3 (CH_3), 51.2 (CH_2), 49.1 (CH_2), 38.2 (CH), 31.8 (2 x CH_2), 27.4 (CH_2), 25.7 (CH_2), 22.4 (2 x CH_2). HR-ESMS calcd. for $C_{24}H_{33}N_2O_3^+$ [M + H] 397.2486, found 397.2484.

Synthesis of (*E*)-((*R*)-1-((1s*,4S*)-4-hydroxycyclohexyl)piperidin-3-yl)(6-methoxynaphthalen-2-yl)methanone O-ethyl oxime (***WEHI-P8***) **(Supplementary Figure 12).**

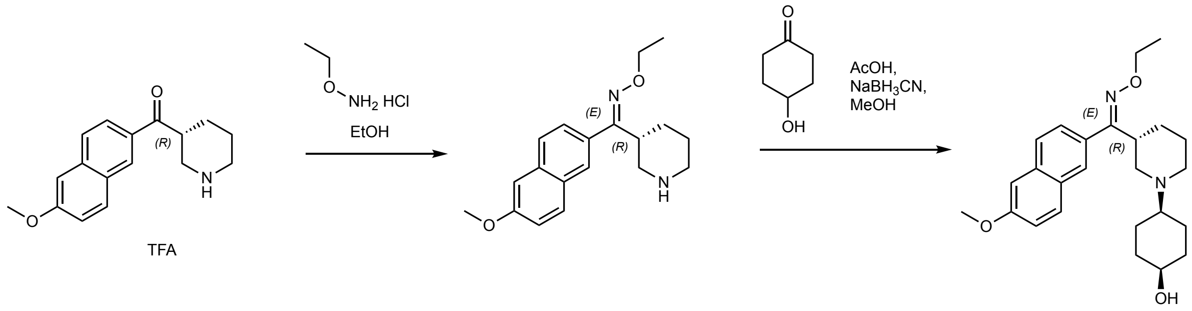

Supplementary Figure 12: Synthesis of **WEHI-P8**

(*R*,*E*)-(6-methoxynaphthalen-2-yl)(piperidin-3-yl)methanone *O*-ethyl oxime

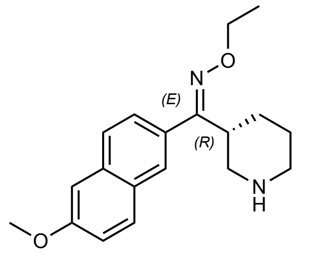

To a stirred solution of (*R*)-(6-methoxynaphthalen-2-yl)(piperidin-3-yl)methanone (TFA salt) (22.0 g, 60.0 mmol) in ethanol (200 mL) under nitrogen atmosphere was added *O*-ethylhydroxylamine hydrochloride (11.7 g, 2 eq., 120 mmol). The reaction mixture was stirred at 90°C for 12h. The reaction mixture was evaporated under reduced pressure, ice cold water added, and the mixture extracted with EtOAc (3 x 100 mL). The combined organics were washed with ice cold water and sat. NaCl, dried over Na_2SO_4, filtered and evaporated under reduced pressure. The crude compound was purified by RP-HPLC purification using Waters X-Bridge C8, 19x250mm, 5µ, 5mM NH_4HCO_3 (+0.1% NH_4OH) in H_2O/MeCN for E/Z separation to obtain Peak 1 (*R*,*E*)-(6-methoxynaphthalen-2-yl)(piperidin-3-yl)methanone *O*-ethyl

oxime (3.2 g, 17%) and Peak 2 (*R*,*Z*)-(6-methoxynaphthalen-2-yl)(piperidin-3-yl)methanone *O*-ethyl oxime (12.0 g, 64%).

(*E*)-((*R*)-1-((1s*,4S*)-4-hydroxycyclohexyl)piperidin-3-yl)(6-methoxynaphthalen-2-yl)methanone *O*-ethyl oxime **(*WEHI-P8*)**

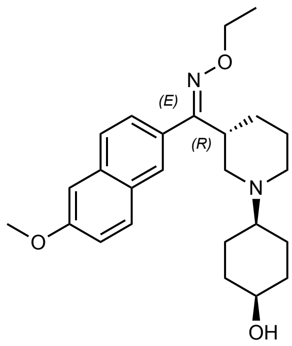

A solution of (*R*,*E*)-(6-methoxynaphthalen-2-yl)(piperidin-3-yl)methanone *O*-ethyl oxime (2.15 g, 6.88 mmol) and 4-hydroxycyclohexanone (2.86 mL, 4 eq., 27.5 mmol) in MeOH (20 mL) at ambient temperature under nitrogen atmosphere was stirred for 5 minutes. Then AcOH (39.4 µL, 0.1 eq., 0.68 mmol) and $NaBH_3CN$ (1.30 g, 3 eq., 20.6 mmol) was added and the reaction mixture stirred at ambient temperature for 16h. The reaction mixture was poured on ice-cold water and extracted with EtOAc (3 x 50 mL). The combined organic layers were washed with sat. NaCl solution, dried over Na_2SO_4, filtered, and evaporated under reduced pressure to give the crude compound (1.45 g). This was purified by RP-HPLC purification using Waters X-Bridge C8, 19x250mm, 5µ, 5mM NH_4HCO_3 (+0.1% NH_4OH) in H_2O/MeCN to afford 700 mg (Peak 1) and 750 mg (Peak 2). Peak 2 was further purified by chiral HPLC using Chiralcel OX-H, 30x250mm, 5µ, 0.1% NH_4OH in MeOH to obtain Peak 1 (*E*)-((*R*)-1-((1s*,4S*)-4-hydroxycyclohexyl)piperidin-3-yl)(6-methoxynaphthalen-2-yl)methanone *O*-ethyl oxime **(*WEHI-P8*)** (285 mg, 10%, >99% purity, >99% ee) and Peak 2 (*E*)-((*S*)-1-((1s*,4S*)-4-hydroxycyclohexyl)piperidin-3-yl)(6-methoxynaphthalen-2-yl)methanone *O*-ethyl oxime (68 mg). ^{1}H NMR (300 MHz, DMSO) δ 7.90 (d, J = 9.0 Hz, 1H), 7.85 – 7.77 (m, 2H), 7.49 (dd, J = 8.5, 1.7 Hz, 1H), 7.33 (d, J = 2.5 Hz, 1H), 7.18 (dd, J = 9.0, 2.5 Hz, 1H), 4.24 (d, J = 3.3 Hz, 1H), 4.13 (q, J = 7.0 Hz, 2H), 3.88 (s,

3H), 3.68 (s, 1H), 3.40 – 3.29 (m, 1H*), 2.90 – 2.77 (m, 2H), 2.39 (t, J = 10.9 Hz, 1H), 2.26 – 2.04 (m, 2H), 1.78 – 1.45 (m, 6H), 1.41 – 1.31 (m, 3H), 1.25 (t, J = 7.0 Hz, 3H). *This signal was observed with the addition of D_2O. ^{13}C NMR (75 MHz, DMSO) δ 160.7 (C), 157.7 (C), 134.0 (C), 131.0 (C), 129.8 (CH), 127.8 (C), 126.7 (CH), 126.3 (CH), 126.0 (CH), 119.0 (CH), 105.8 (CH), 68.8 (CH_2), 64.2 (CH), 62.2 (CH), 55.2 (CH_3), 51.1 (CH_2), 49.2 (CH_2), 38.2 (CH), 31.8 (2 x CH_2), 27.3 (CH_2), 25.7 (CH_2), 22.6 (CH_2), 22.4 (CH_2), 14.7 (CH_3). HR-ESMS calcd. for $C_{25}H_{35}N_2O_3^+$ [M + H] 411.2642, found 411.2641.

(1-(4-Hydroxycyclohexyl)piperidin-3-yl)(6-methoxynaphthalen-2-yl)methanone (formic acid salt) (***WEHI-P24***)

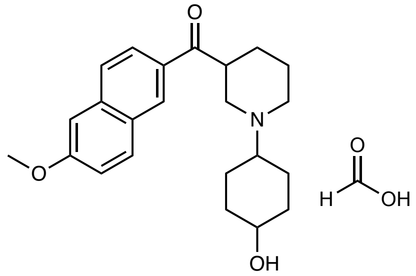

To a stirred solution of (6-methoxynaphthalen-2-yl)(piperidin-3-yl)methanone (HCl salt) (250 mg, 0.82 mmol) and 4-hydroxycyclohexanone (187 mg, 1 eq., 1.64 mmol) in DCE (2 mL) at ambient temperature under nitrogen atmosphere was added $NaBH(OAc)_3$ (343 mg, 2.0 eq., 1.64 mmol). The reaction mixture was stirred at ambient temperature for 3h. The reaction mixture was poured on ice-cold water and extracted with EtOAc (3 x 30 mL). The combined organics were washed with sat. NaCl solution, dried over Na_2SO_4, filtered, and evaporated under reduced pressure to give the crude compound. This was purified by RP-HPLC using Waters X-Bridge C18, 19x250mm, 10µ, 0.1% FA in H_2O/MeCN to afford (1-(4-hydroxycyclohexyl)piperidin-3-yl)(6-methoxynaphthalen-2-yl)methanone as a formic acid salt (10 mg, 3.3%, >99% purity). ^1H NMR (300 MHz, DMSO) δ 8.59 (s, 1H), 8.22 (s, 1H), 8.06 (d, J = 9.0 Hz, 1H), 7.94 – 7.88 (m, 2H), 7.40 (d, J = 2.5 Hz, 1H), 7.26 (dd, J =

8.9, 2.5 Hz, 1H), 3.91 (s, 3H), 3.80 – 3.68 (m, 3H), 3.10 – 2.79 (m, 2H), 2.46 – 2.19 (m, 3H), 1.96 – 1.56 (m, 7H), 1.54 – 1.03 (m, 5H). ^{13}C NMR (75 MHz, DMSO) δ 201.5 (C), 201.4 (C), 163.8 (C), 159.4 (C), 136.9 (C), 131.4 (CH), 130.9 (C), 129.8 (CH), 127.6 (C), 127.2 (CH), 124.5 (CH), 119.5 (CH), 106.0 (CH), 68.7 (CH), 63.8 (CH), 62.9 (CH), 62.8 (CH), 55.5 (CH_3), 52.0 (CH_2), 51.7 (CH_2), 49.1 (CH_2), 43.6 (CH), 34.5 (CH_2), 31.8 (CH_2), 31.7 (CH_2), 28.0 (CH_2), 27.9 (CH_2), 25.7 (CH_2), 25.3 (CH_2), 24.7 (CH_2), 24.5 (CH_2), 22.0 (CH_2), 21.8 (CH_2). HR-ESMS calcd. for $C_{23}H_{30}NO_3^+$ [M + H] 368.2220, found 368.2217.

Synthesis (*E*)-((*R*)-1-((1s*,4S*)-4-(1*H*-pyrazol-3-yl)cyclohexyl)piperidin-3-yl)(6-methoxynaphthalen-2-yl)methanone *O*-methyl oxime (***WEHI-P70***) **(Supplementary Figure 13).**

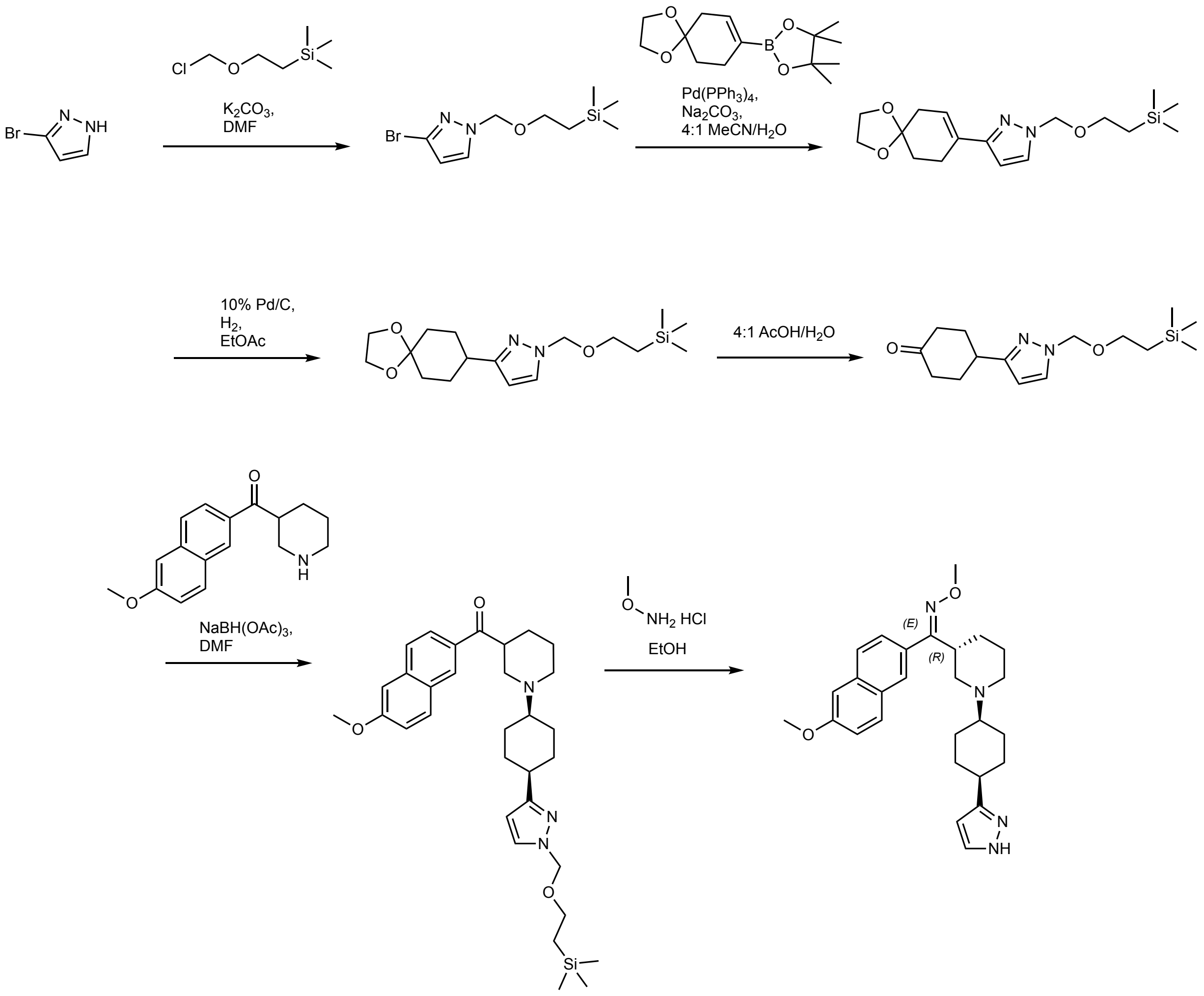

Supplementary Figure 13: *Synthesis of* ***WEHI-P70***

3-Bromo-1-((2-(trimethylsilyl)ethoxy)methyl)-1*H*-pyrazole

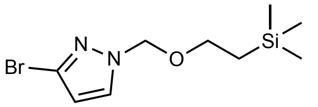

To a stirred solution of 3-bromopyrazole (6.0 g, 40.8 mmol) in DMF (20 mL) was added K_2CO_3 (8.46, 1.5 eq., 61.2 mmol). The mixture was cooled to 0°C and 2-(trimethylsilyl)ethoxymethyl chloride (7.95 mL, 1.1 eq., 44.9 mmol) was added dropwise and the reaction stirred at ambient temperature for 16h. The reaction mixture was poured on ice-cold water and extracted with EtOAc (3 x 80 mL). The combined organic layers were washed with sat. NaCl solution, dried over

Na_2SO_4, filtered, and evaporated under reduced pressure, and purified by SiO_2 column chromatography (30% EtOAc in heptane) to afford 3-bromo-1-((2-(trimethylsilyl)ethoxy)methyl)-1H-pyrazole (5.5 g, 44%) as a colourless oil.

3-(1,4-Dioxaspiro[4.5]dec-7-en-8-yl)-1-((2-(trimethylsilyl)ethoxy)methyl)-1*H*-pyrazole

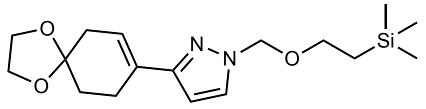

To a solution of 3-bromo-1-((2-(trimethylsilyl)ethoxy)methyl)-1H-pyrazole (5.3 g, 19.1 mmol), 1,4-dioxaspiro[4.5]dec-7-en-8-boronic acid pinacol ester (6.61 g, 1.3 eq., 24.9 mmol) and Na_2CO_3 (3.04 g, 1.5 eq., 28.7 mmol) in a 4:1 mixture of MeCN/H_2O (80 mL) was added $Pd(PPh_3)_4$ (2.21 g, 1.91 mmol). The mixture was degassed with nitrogen for 10 min then heated at 100 °C for 12h. The reaction mixture was filtered through a Celite® pad and the filtrate extracted with EtOAc (3 x 80 mL). The combined organic layers were washed with sat. NaCl solution, dried over Na_2SO_4, filtered, and evaporated under reduced pressure, and purified by column chromatography (30% EtOAc in heptane) to afford 3-(1,4-dioxaspiro[4.5]dec-7-en-8-yl)-1-((2-(trimethylsilyl)ethoxy)methyl)-1H-pyrazole (4.5 g, 52%) as a colourless oil.

3-(1,4-Dioxaspiro[4.5]decan-8-yl)-1-((2-(trimethylsilyl)ethoxy)methyl)-1*H*-pyrazole

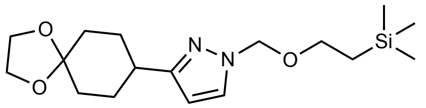

To a solution of 3-(1,4-dioxaspiro[4.5]dec-7-en-8-yl)-1-((2-(trimethylsilyl)ethoxy)methyl)-1*H*-pyrazole (6.3 g, 18.7 mmol) in EtOAc (80 mL) under nitrogen atmosphere was added 10% Pd/C (5.0 g, 2.5eq., 47 mmol). The flask was evacuated and backfilled with hydrogen and the reaction stirred at ambient temperature for 16h. The reaction mixture was filtered through a Celite® pad and the filtrate concentrated under reduced pressure to afford the

crude 3-(1,4-dioxaspiro[4.5]decan-8-yl)-1-((2-(trimethylsilyl)ethoxy)methyl)-1*H*-pyrazole (6.0 g, 94%) as a colourless oil used without further purification.

4-(1-((2-(Trimethylsilyl)ethoxy)methyl)-1*H*-pyrazol-3-yl)cyclohexanone

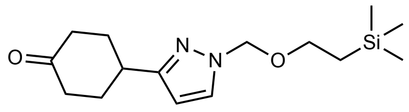

A solution of 3-(1,4-dioxaspiro[4.5]decan-8-yl)-1-((2-(trimethylsilyl)ethoxy)methyl)-1*H*-pyrazole (5.9 g, 17.4 mmol) was stirred in a 4:1 mixture of AcOH/H_2O (25 mL) at ambient temperature for 12 h. The reaction was quenched with sat. $NaHCO_3$ solution and extracted with EtOAc (3 x 50 mL). The combined extracts were dried over Na_2SO_4, filtered, and evaporated under reduced pressure to afford crude 4-(1-((2-(trimethylsilyl)ethoxy)methyl)-1*H*-pyrazol-3-yl)cyclohexanone (3.3 g, 55%) as a sticky yellow oil used without further purification.

(6-Methoxynaphthalen-2-yl)(1-(4-(1-((2-(trimethylsilyl)ethoxy)methyl)-1*H*-pyrazol-3-yl)cyclohexyl)piperidin-3-yl)methanone

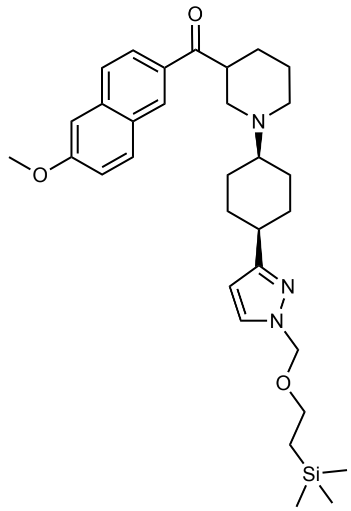

A solution of 4-(1-((2-(trimethylsilyl)ethoxy)methyl)-1*H*-pyrazol-3-yl)cyclohexanone (3.15 g, 10.7 mmol) and (6-methoxynaphthalen-2-yl)(piperidin-3-yl)methanone (2.4 g, 8.91 mmol) in DMF (30 mL) was stirred at ambient temperature for 1h after which $NaBH(OAc)_3$ (5.67 g, 3.0 eq., 26.7 mmol) was added. The reaction was stirred at ambient temperature for 12h. The reaction mixture was poured on ice-cold water and extracted with DCM (3 x 80 mL). The combined organic layers were washed with sat. NaCl solution, dried over Na_2SO_4, filtered, and evaporated under reduced pressure, and purified by RP-HPLC using Waters X-Bridge C8, 19x250mm, 10µ, 0.1% NH_4OH in H_2O/MeCN to obtain Peak 1 (6-methoxynaphthalen-2-yl)(1-((1r*,4R*)-4-(1-((2-(trimethylsilyl)ethoxy)methyl)-1H-pyrazol-3-yl)cyclohexyl)piperidin-3-yl)methanone (1.05 g, 21%) and Peak 2 (6-methoxynaphthalen-2-yl)(1-((1s*,4S*)-4-(1-((2-(trimethylsilyl)ethoxy)methyl)-1H-pyrazol-3-yl)cyclohexyl)piperidin-3-yl)methanone (1.2 g, 24%).

(*E*)-((*R*)-1-((1s*,4S*)-4-(1*H*-pyrazol-3-yl)cyclohexyl)piperidin-3-yl)(6-methoxynaphthalen-2-yl)methanone *O*-methyl oxime **(*WEHI-P70*)**

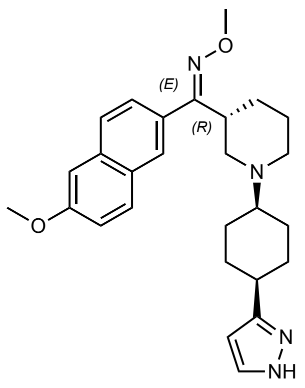

To a solution of (6-methoxynaphthalen-2-yl)(1-((1s*,4S*)-4-(1-((2-(trimethylsilyl)ethoxy)methyl)-1H-pyrazol-3-yl)cyclohexyl)piperidin-3-yl)methanone (500 mg, 913 µmol) in EtOH (5 mL) was added *O*-methylhydroxylamine hydrochloride (457 mg, 6 eq., 5.48 mmol) under nitrogen atmosphere and the reaction mixture was stirred at 90°C for 12h. The reaction mixture was evaporated under reduced pressure, poured onto water and extracted with DCM (2 x 50 mL). The combined organics layers were washed with brine solution, dried over Na_2SO_4, filtered and evaporated under reduced pressure to give the crude compound. This was purified by RP-HPLC using Xtimate Phenyl Hexyl, 19x250mm, 10µ, 0.1% NH_4OH in H_2O/MeCN to give 110 mg (Peak 1) and 65 mg (Peak 2). Peak 1 was further purified by chiral HPLC using Chiralcel-OJ-H (30 mm x 250 mm), 5µ, 0.1% NH_4OH in MeOH to obtain Peak 2 (E)-(1-((1s*,4S*)-4-(1H-pyrazol-3-yl)cyclohexyl)piperidin-3-yl)(6-methoxynaphthalen-2-yl)methanone O-methyl oxime (***WEHI-P70***) (23 mg, 5.6%, >99% purity, >99% ee) as a white solid. ^{1}H NMR (300 MHz, DMSO) δ 12.32 (s, 1H), 7.89 (d, J = 9.0 Hz, 1H), 7.85 – 7.76 (m, 2H), 7.48 (dd, J = 8.5, 1.8 Hz, 1H), 7.44 (d, J = 1.9 Hz, 1H), 7.33 (d, J = 2.5 Hz, 1H), 7.18 (dd, J = 8.9, 2.5 Hz, 1H), 6.04 (d, J = 1.9 Hz, 1H), 3.88 (s, 3H), 3.86 (s, 3H), 3.39 – 3.28 (m, 1H*), 2.92 – 2.78 (m, 3H), 2.31 – 2.18 (m, 2H), 2.02 – 1.86 (m, 3H), 1.75 – 1.41 (m, 10H). *This signal was observed with the addition of D_2O. ^{13}C NMR (75 MHz, DMSO) δ 161.0 (C), 157.8 (C), 134.1 (C), 130.6 (C), 129.8 (CH), 127.8 (C), 126.7 (CH), 126.4 (CH), 125.9

(CH), 119.0 (CH), 105.8 (CH), 102.0 (CH), 61.5 (CH_3), 60.8 (CH), 55.3 (CH_3), 51.6 (CH_2), 49.2 (CH_2), 38.0 (CH), 32.5 (CH), 28.0 (CH_2), 27.9 (CH_2), 27.2 (CH_2), 25.7 (CH_2), 25.5 (CH_2), 25.3 (CH_2). Two carbons (C and CH) not observed. HR-ESMS calcd. for $C_{27}H_{35}N_4O_2^+$ [M + H] 447.2755, found 447.2753.

NMR Data

^{1}H NMR of ***WEHI-P1***

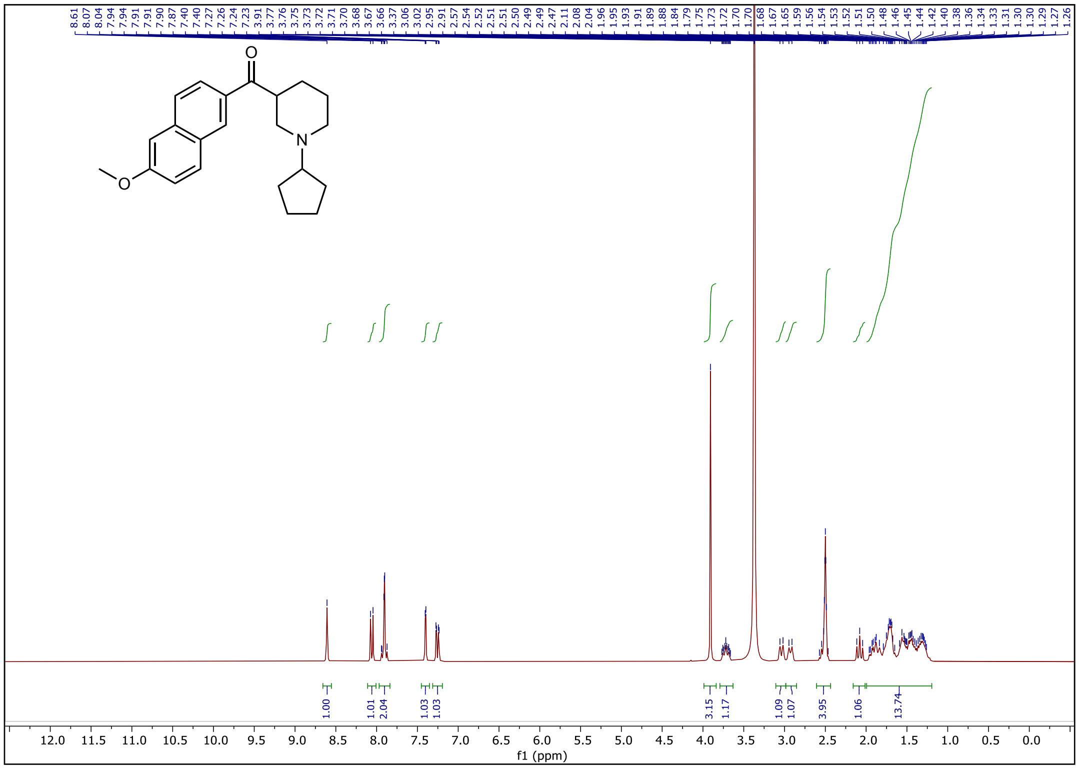

^{1}H NMR (+D_2O) of ***WEHI-P1***

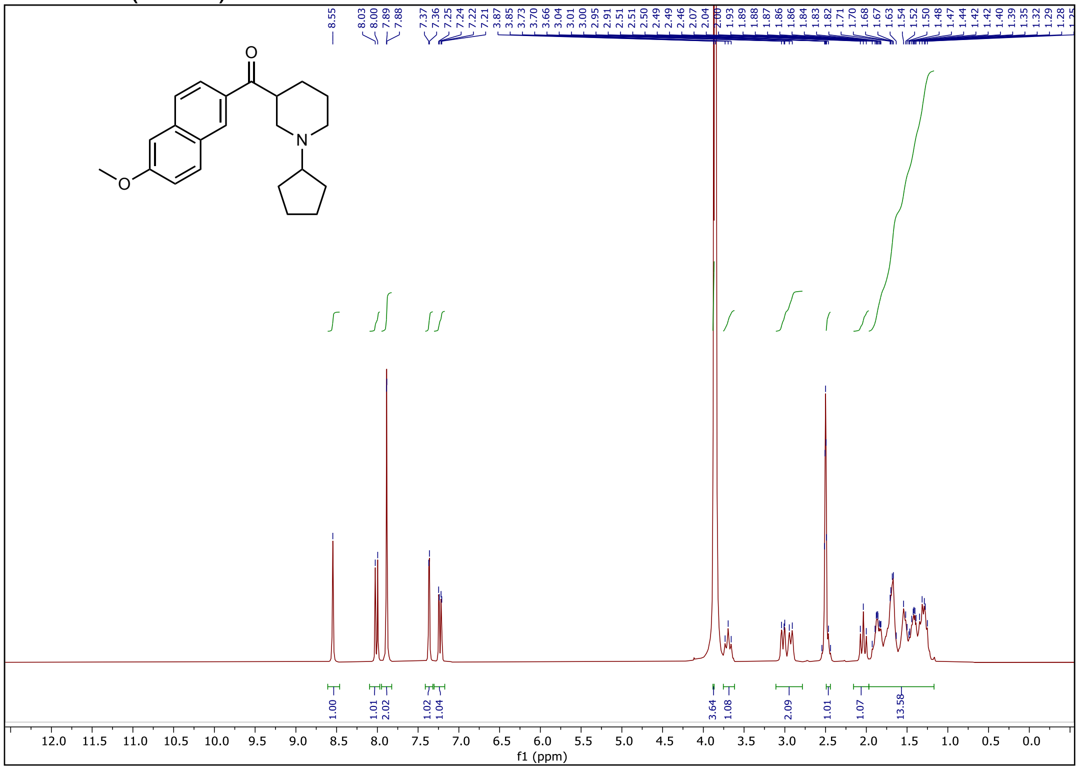

^{13}C NMR of ***WEHI-P1***

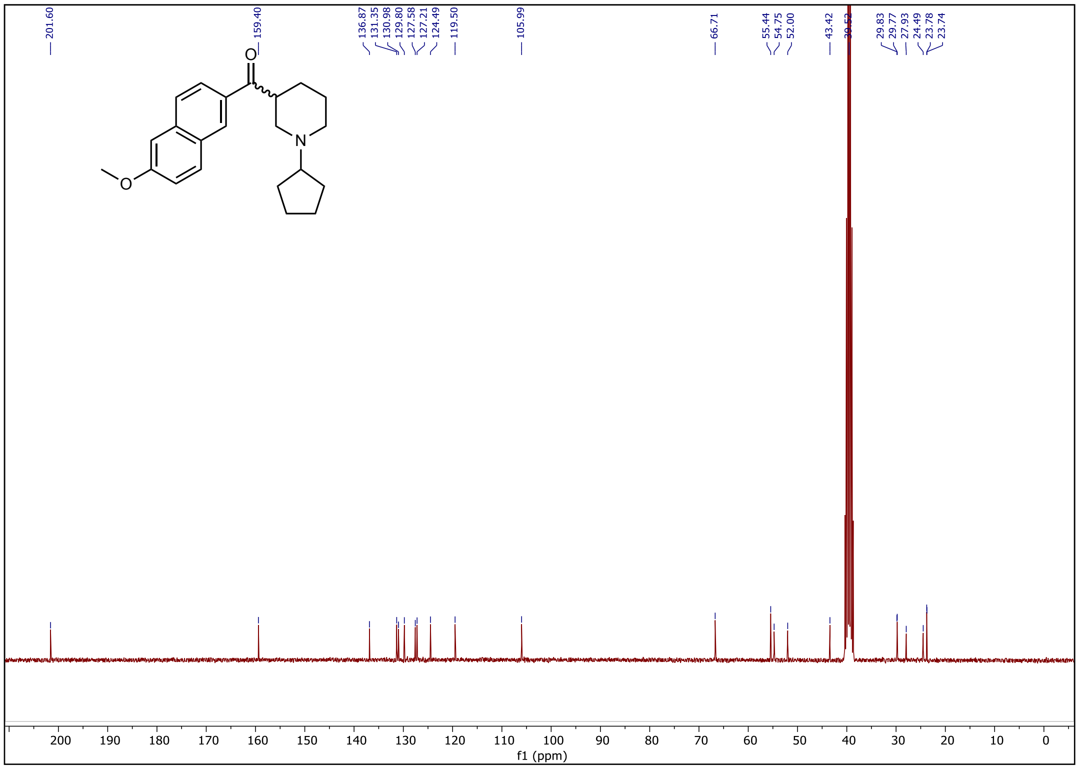

^{13}C DEPTQ NMR of ***WEHI-P1***

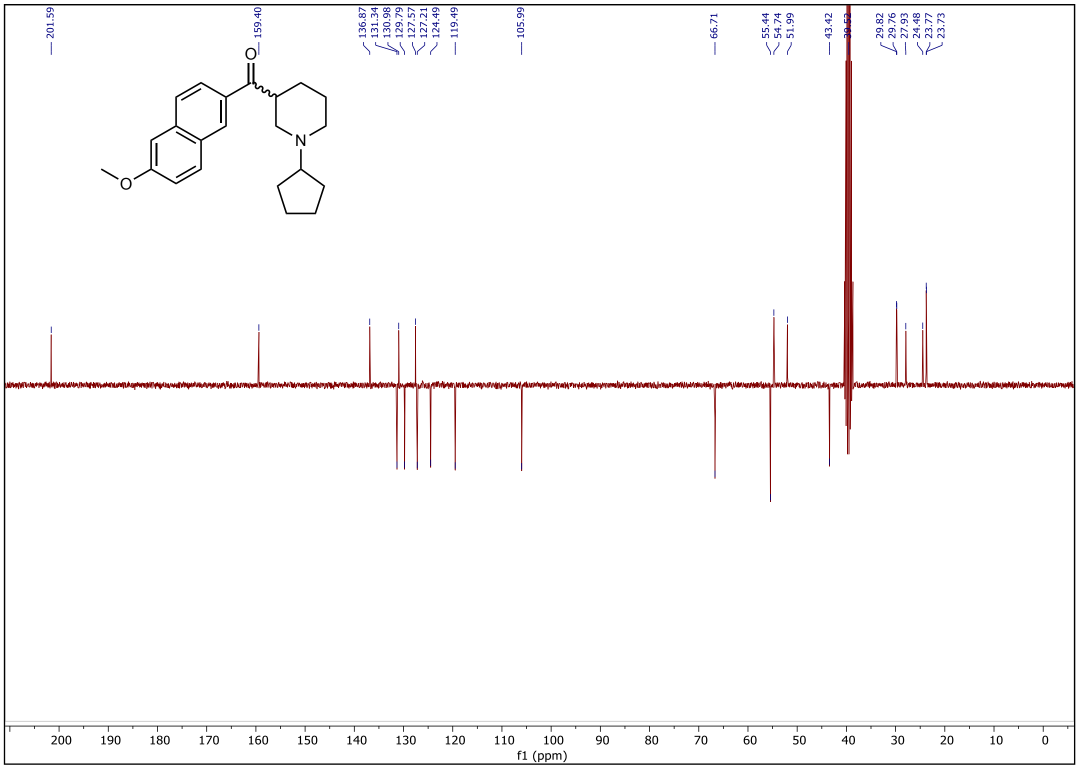

^{1}H NMR of ***WEHI-P2***

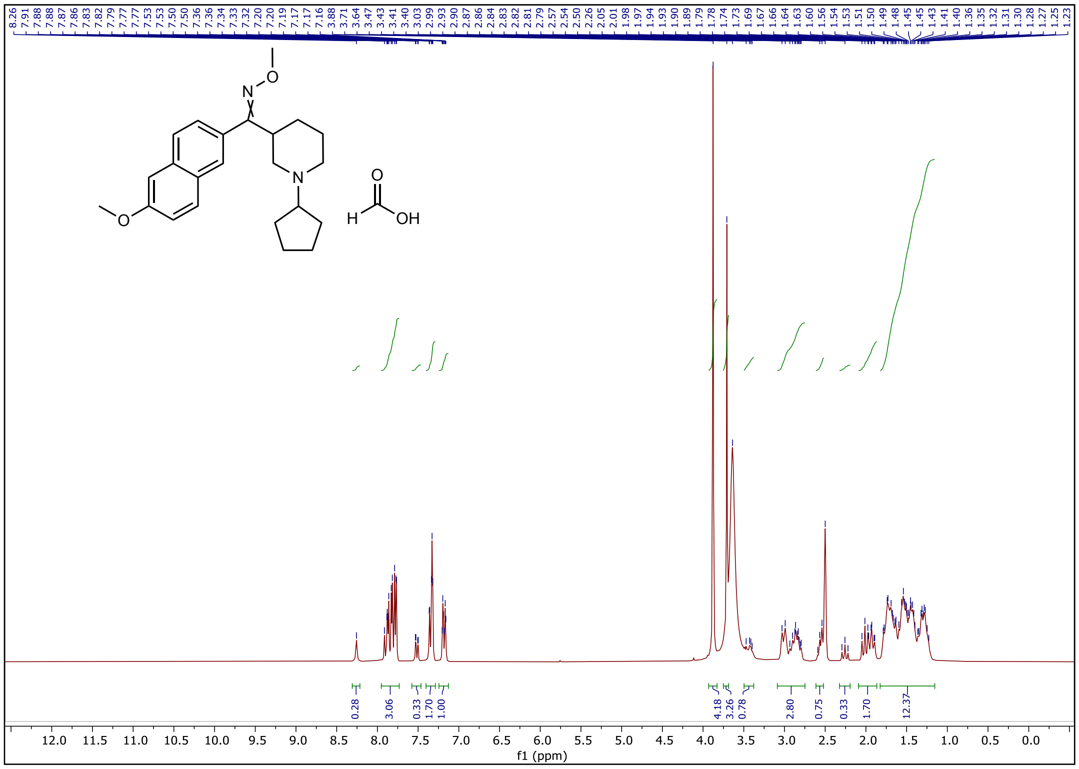

^{1}H NMR (+D_2O) of ***WEHI-P2***

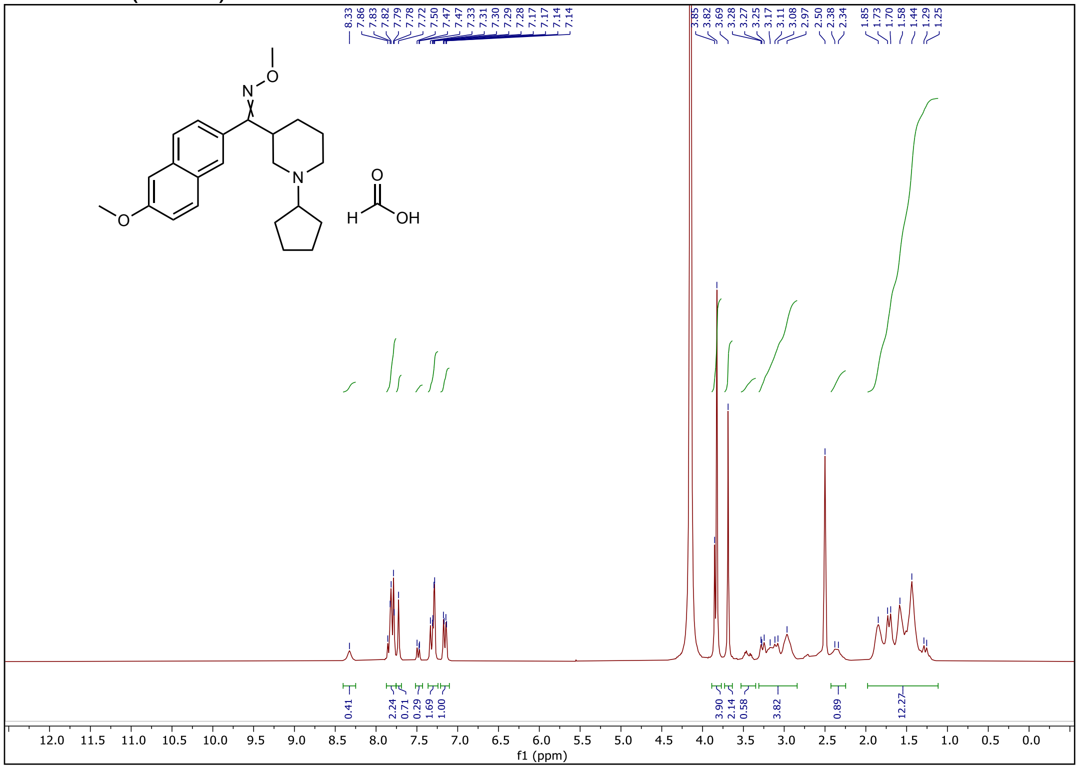

^{13}C NMR of ***WEHI-P2***

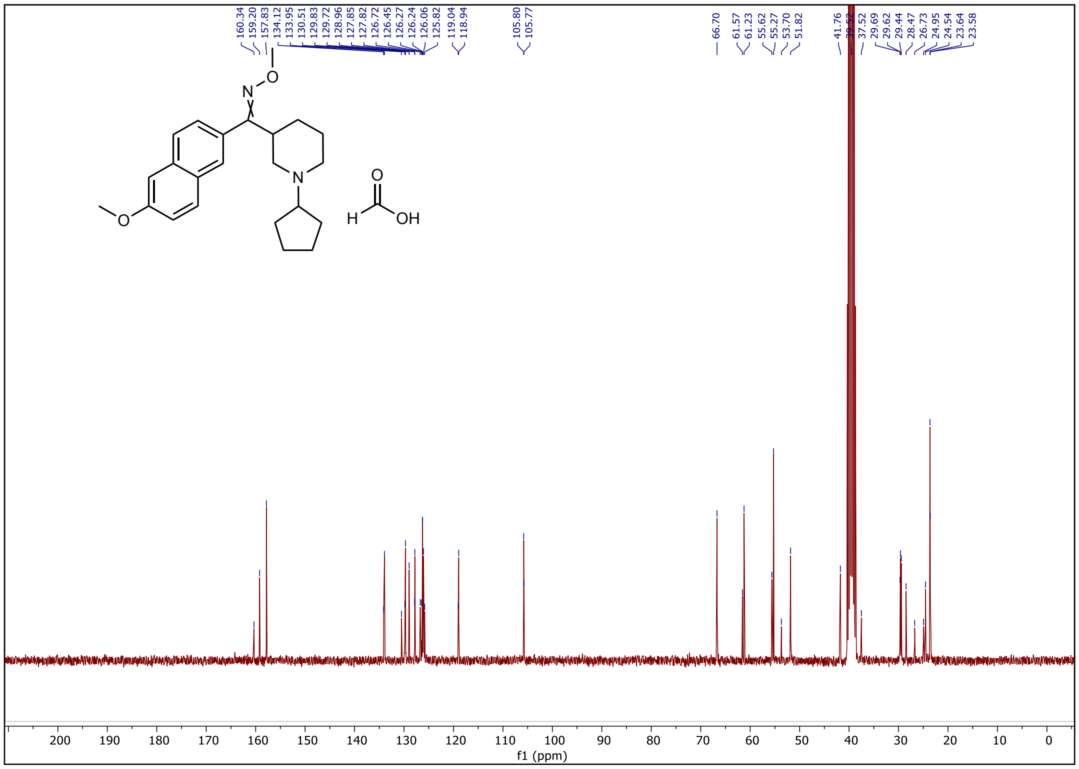

^{13}C DEPTQ NMR of ***WEHI-P2***

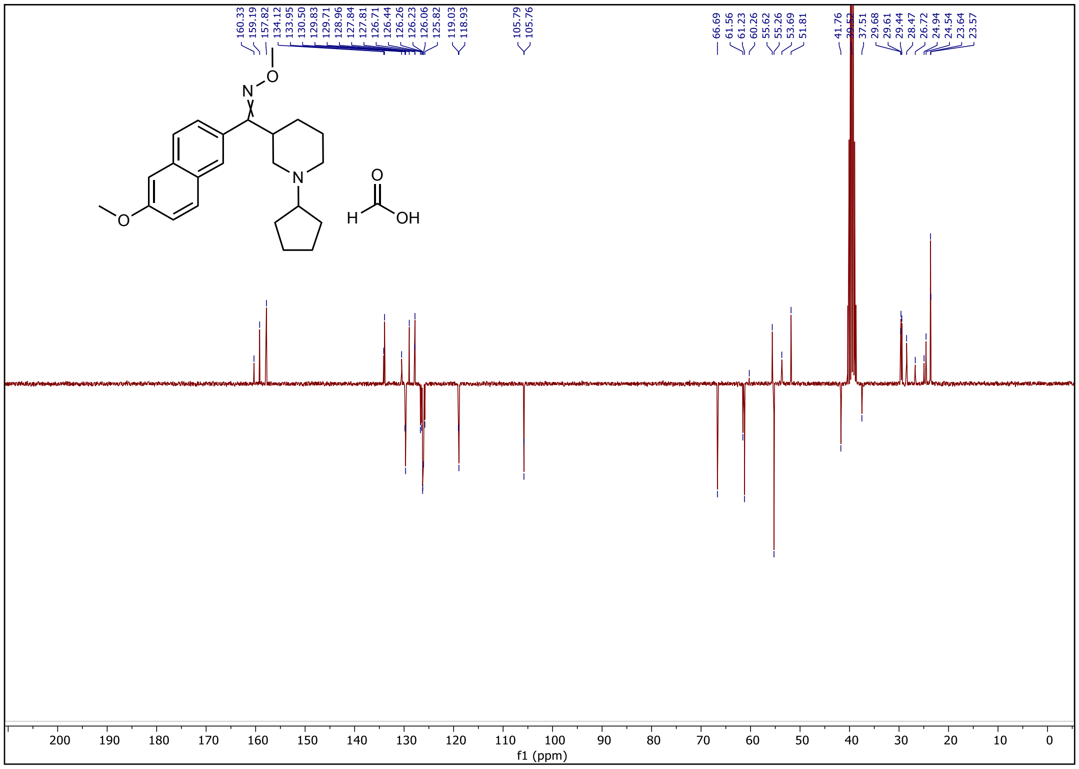

^{1}H NMR of ***WEHI-P4***

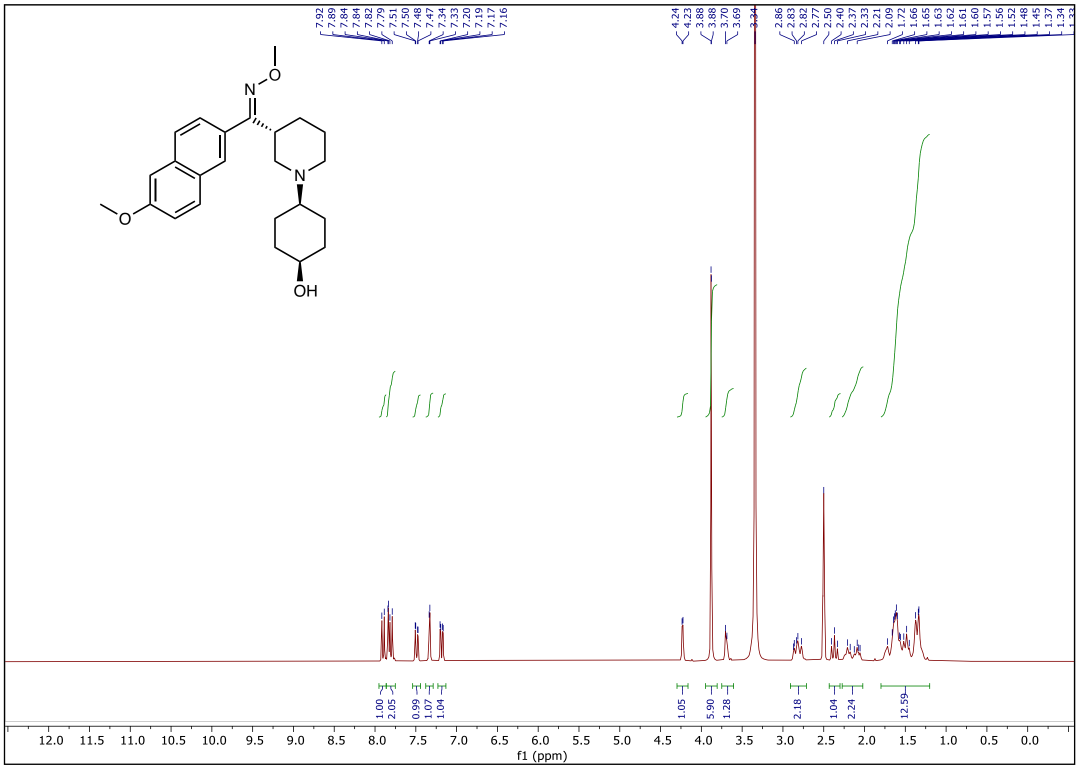

^{1}H NMR (+D_2O) of ***WEHI-P4***

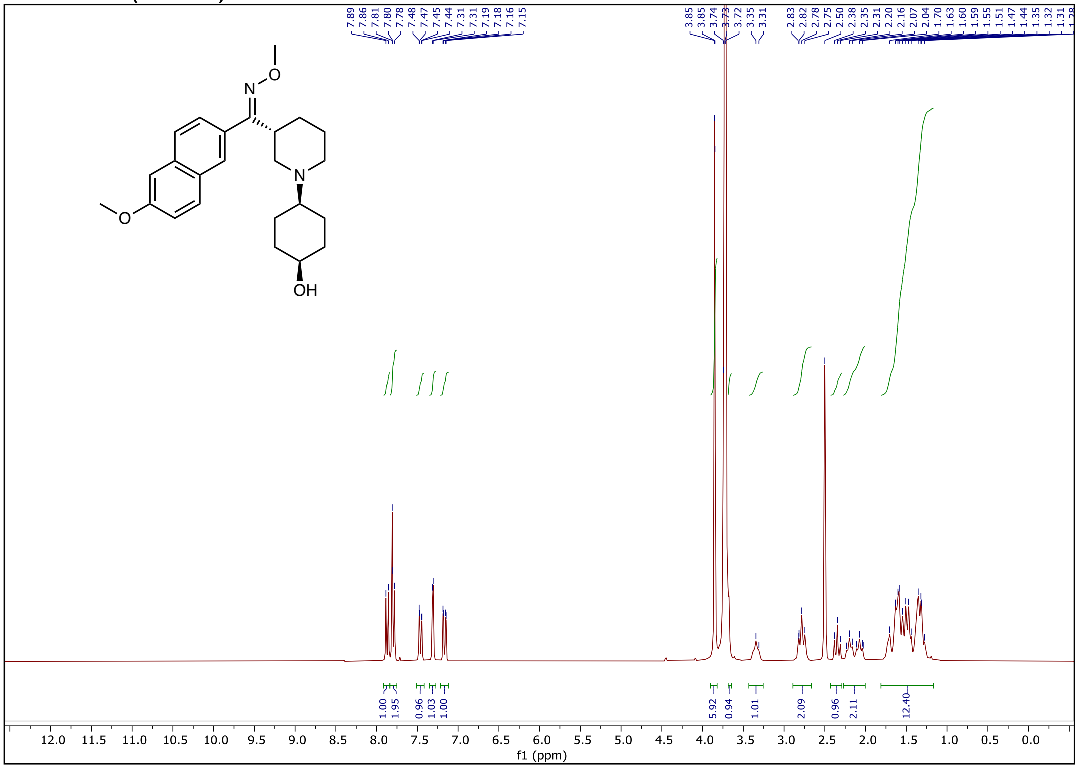

^{13}C NMR of ***WEHI-P4***

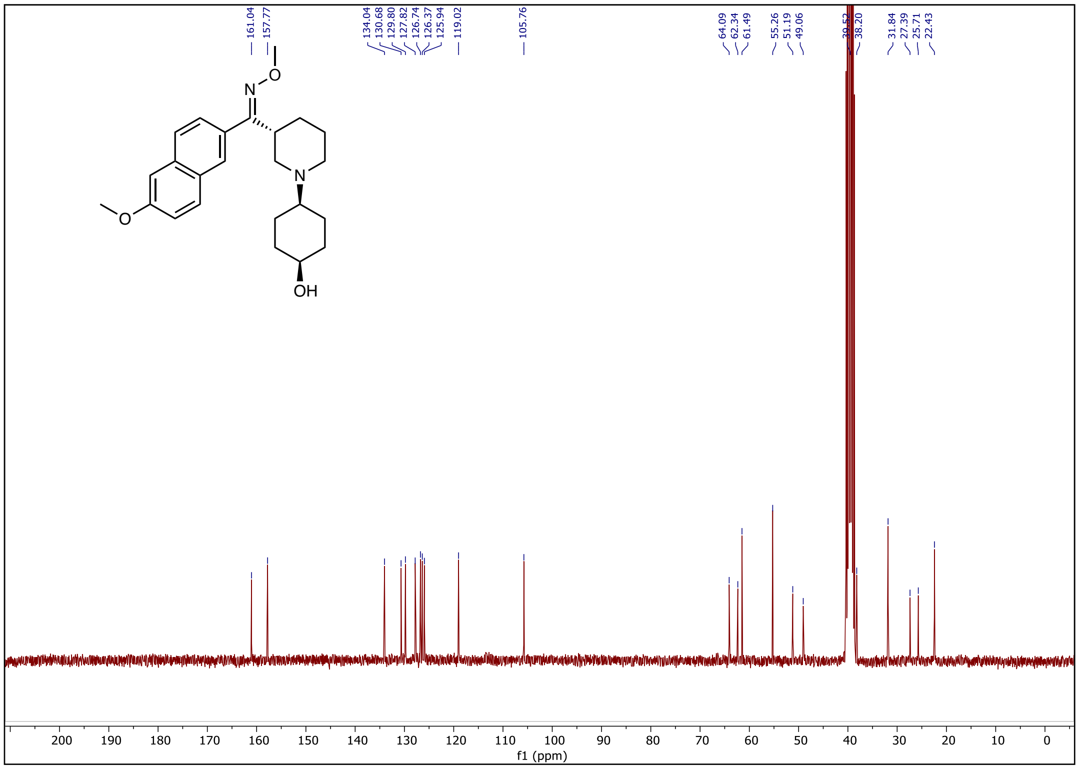

^{13}C DEPTQ NMR of ***WEHI-P4***

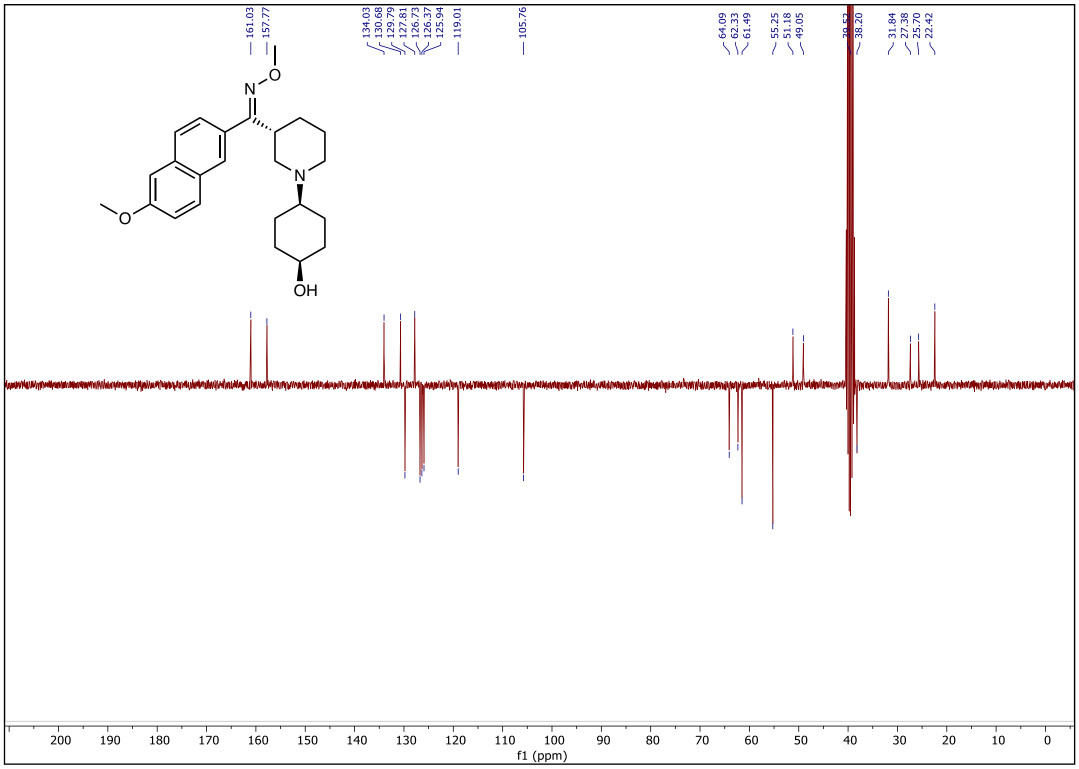

^{1}H NMR of ***WEHI-P8***

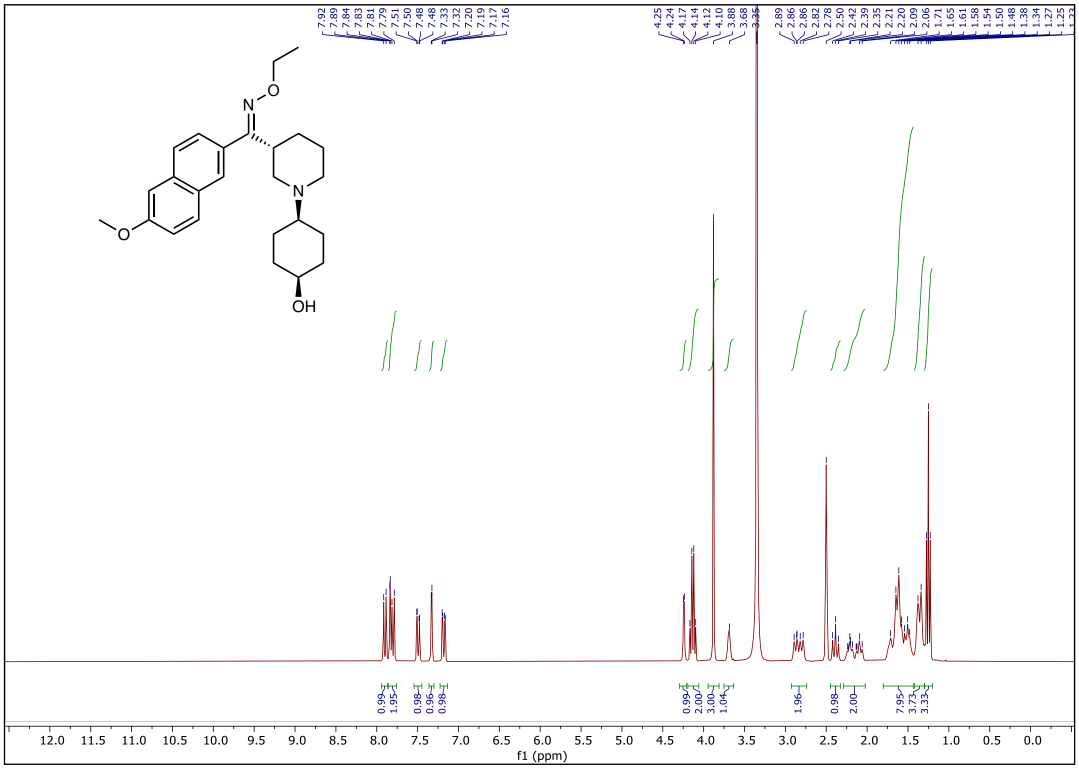

^{1}H NMR (+D_2O) of ***WEHI-P8***

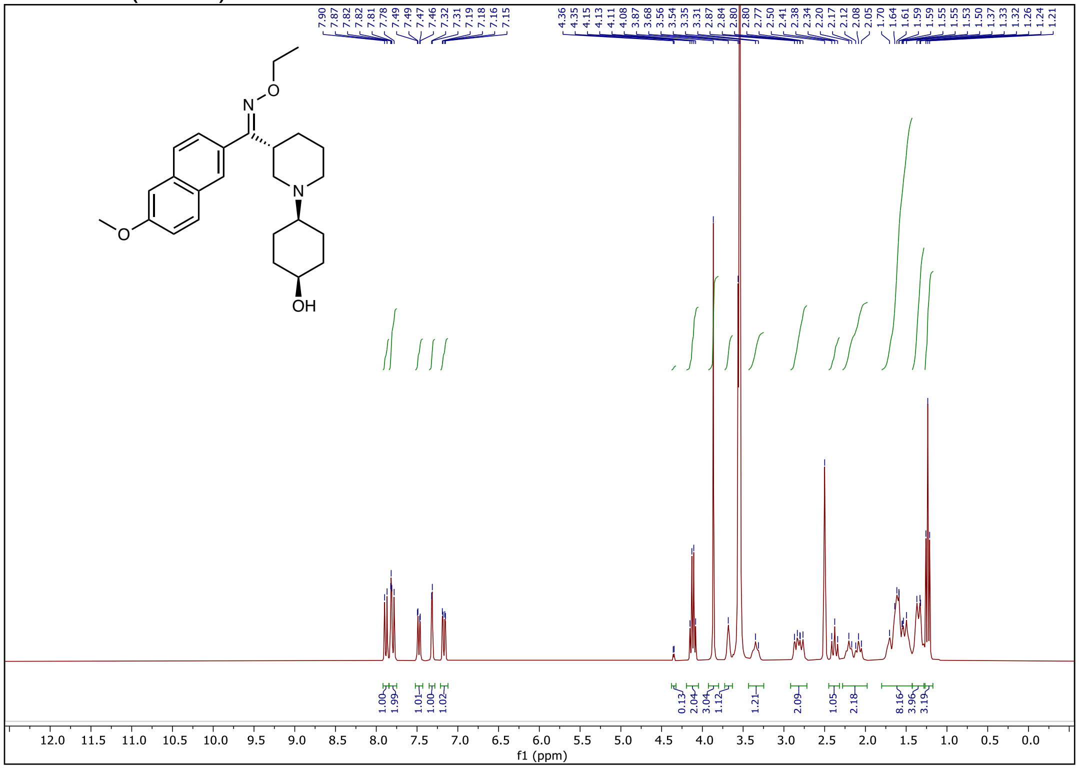

^{13}C NMR of ***WEHI-P8***

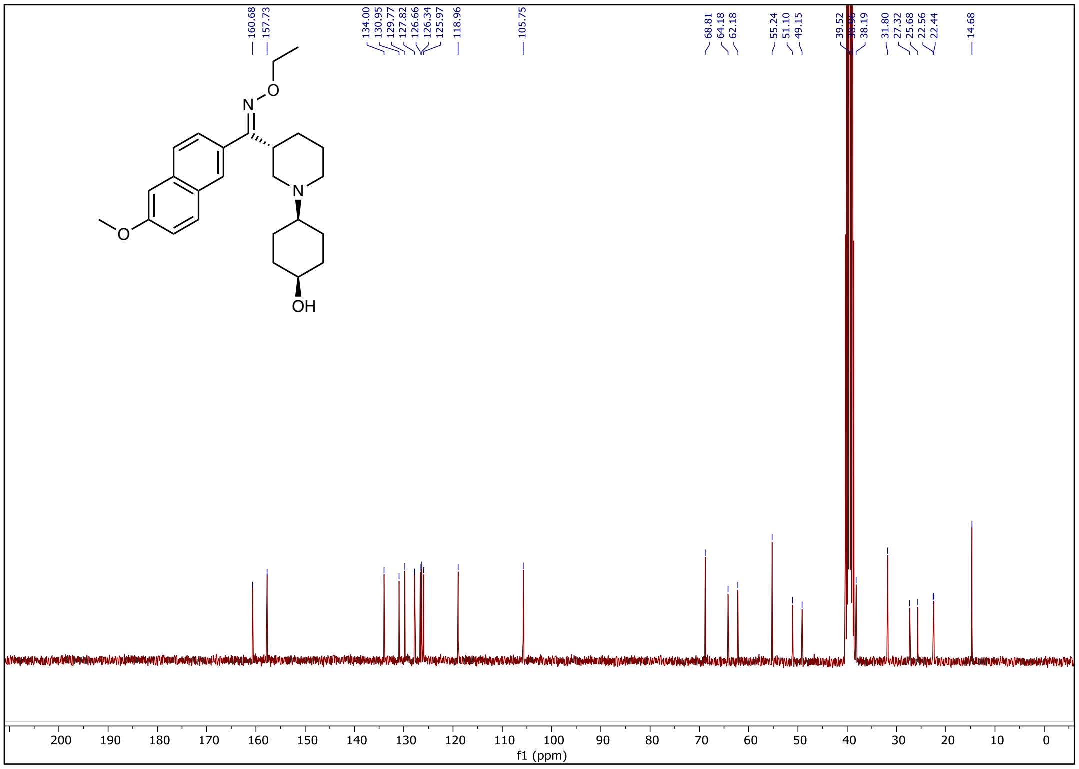

^{13}C DEPTQ NMR of ***WEHI-P8***

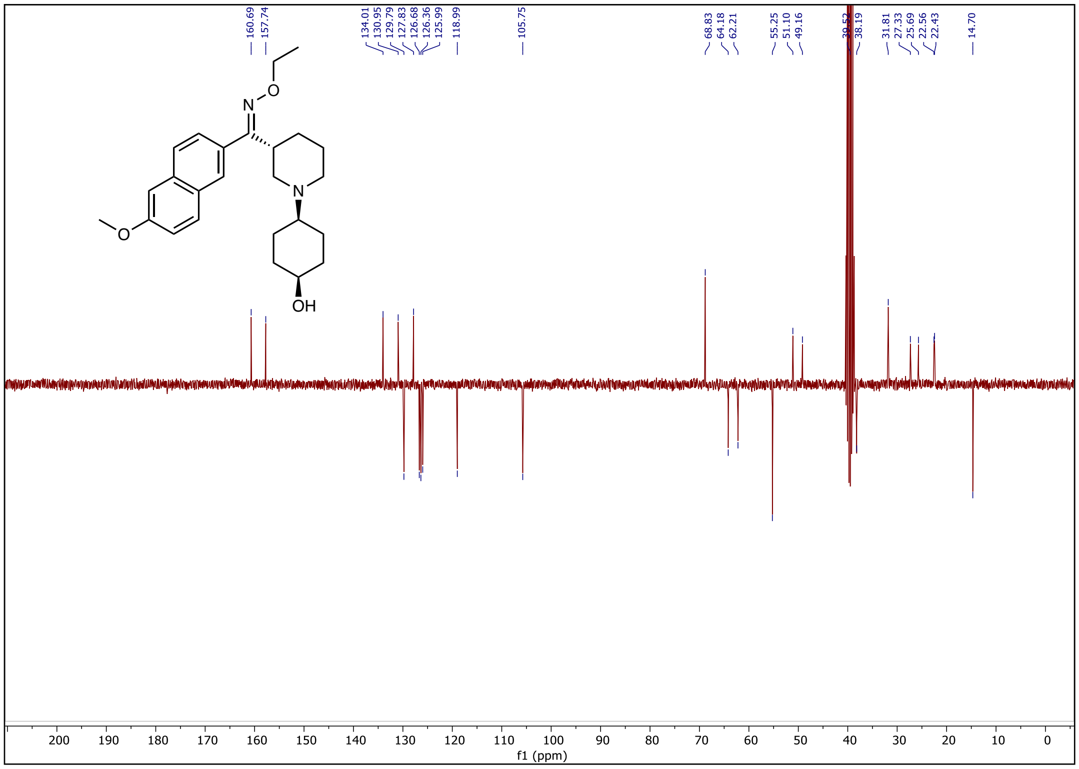

^{1}H NMR of ***WEHI-P24***

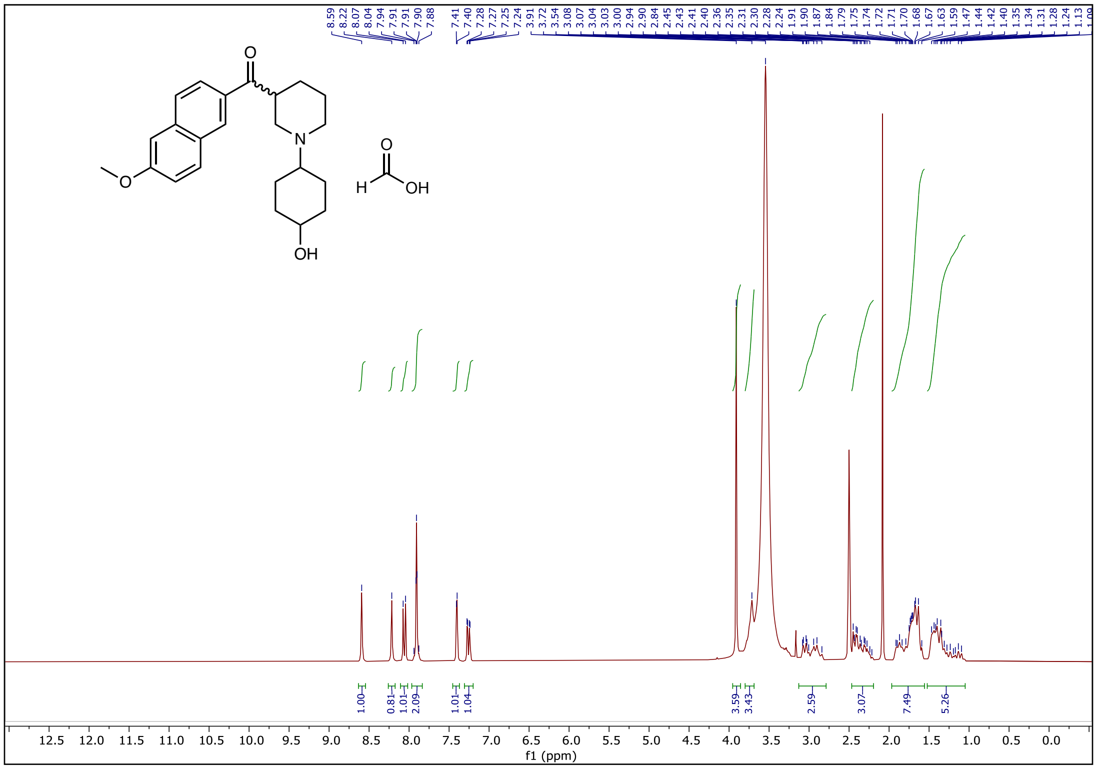

^{1}H NMR (+D_2O) of ***WEHI-P24***

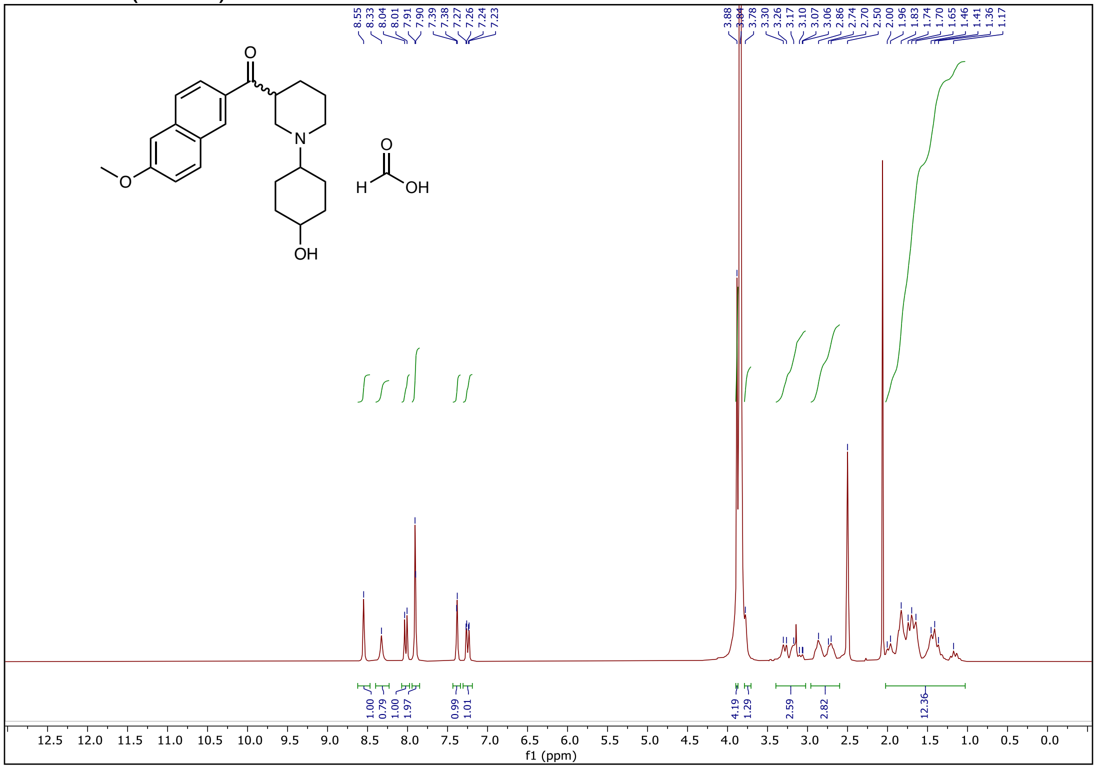

^{13}C NMR of ***WEHI-P24***

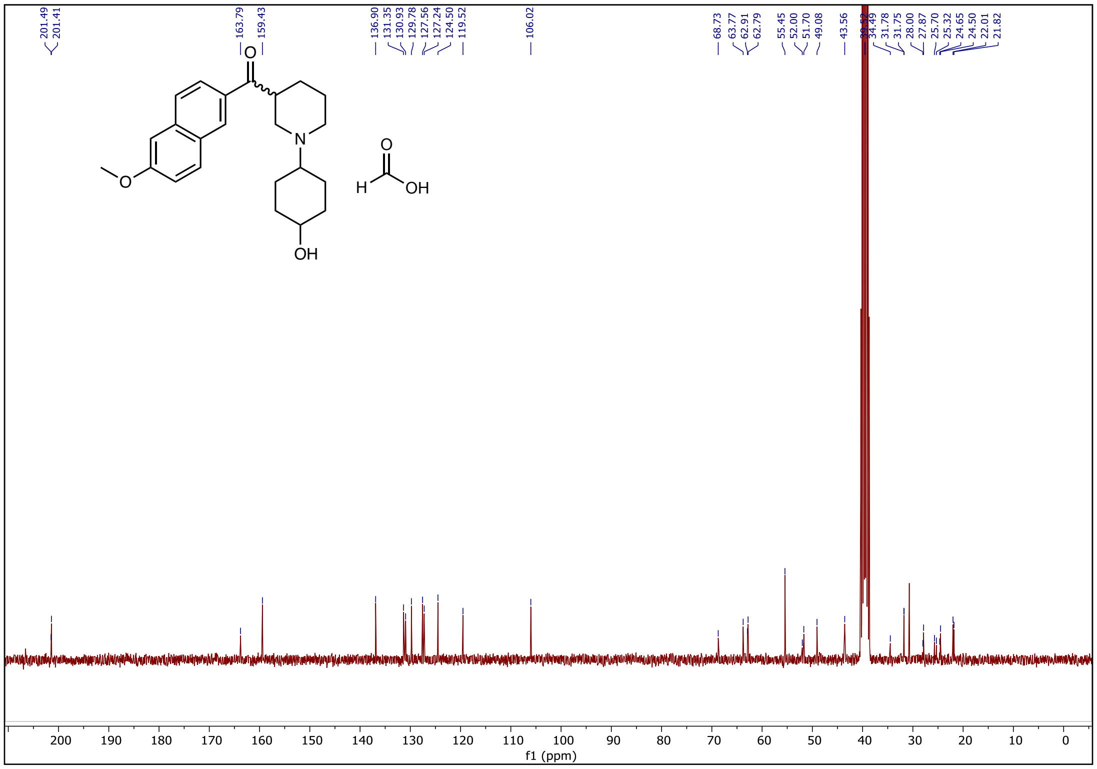

^{13}C DEPTQ NMR of ***WEHI-P24***

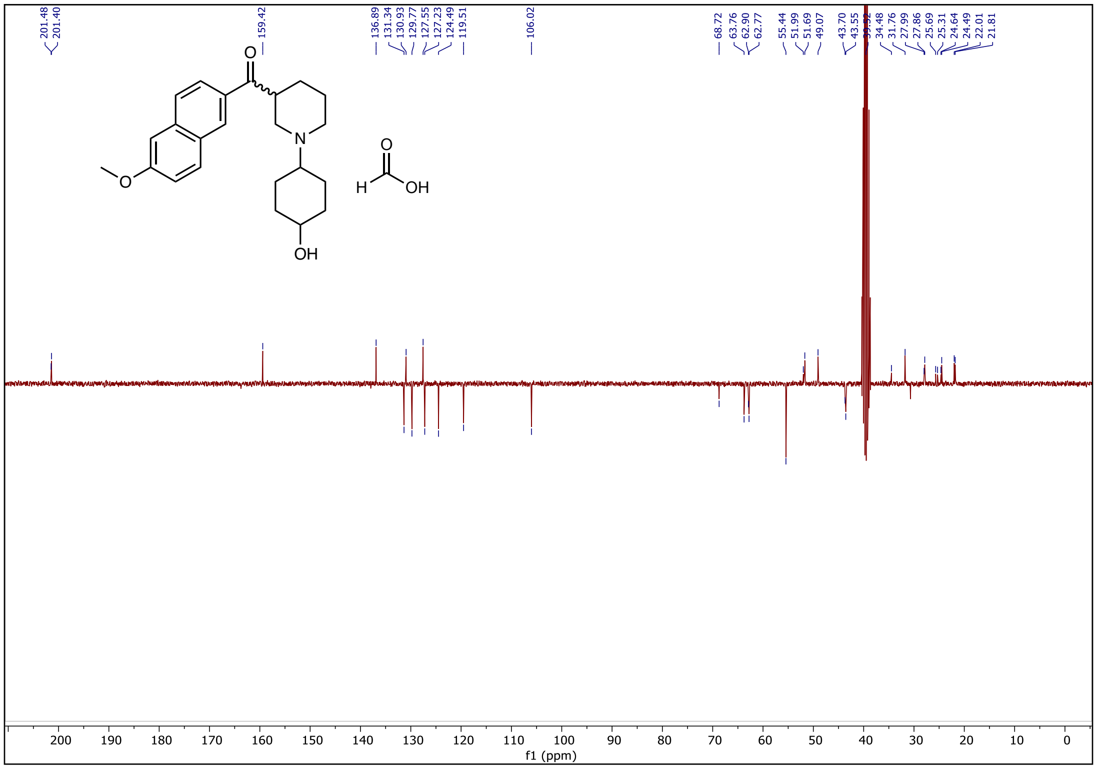

^{1}H NMR of ***WEHI-P70***

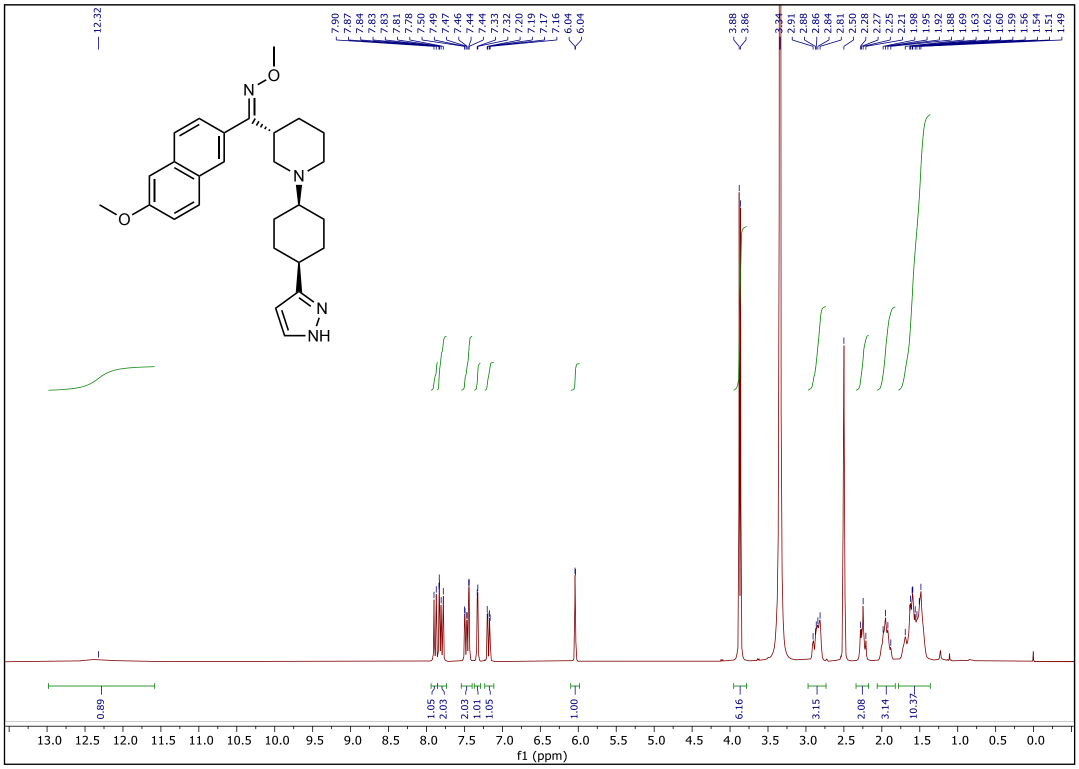

^{1}H NMR (+D_2O) of ***WEHI-P70***

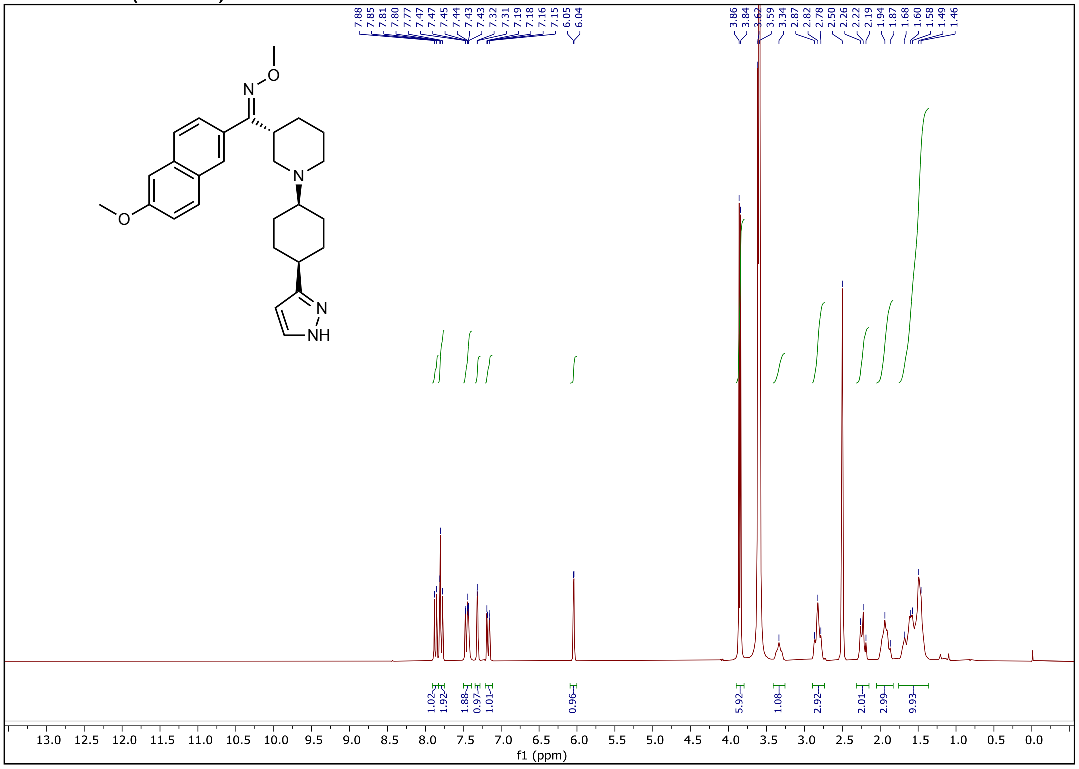

^{13}C NMR of ***WEHI-P70***

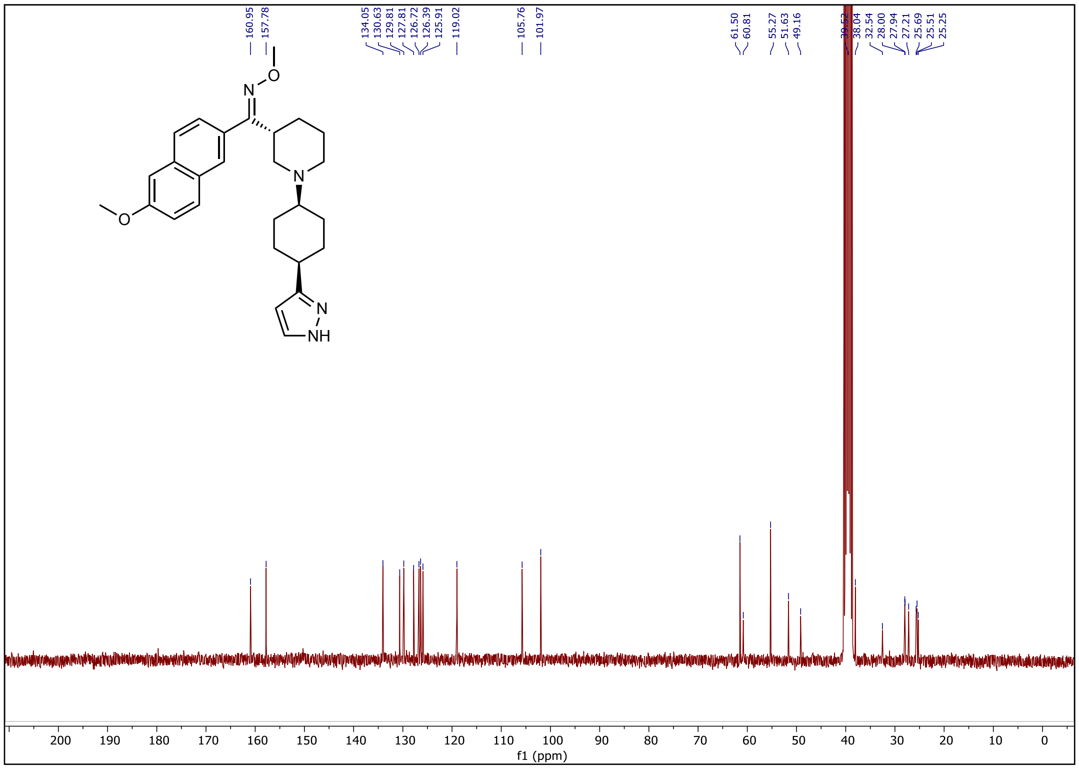

^{13}C DEPTQ NMR of ***WEHI-P70***

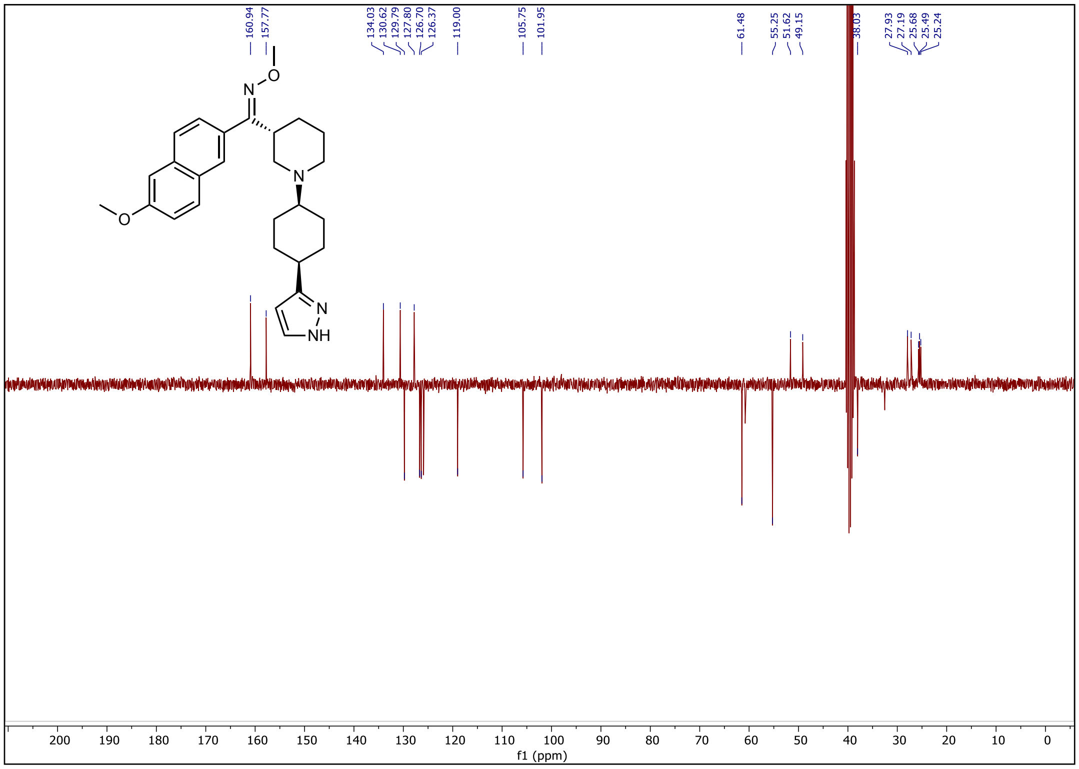

CYP Inhibition

Compound	IC$_{50}$ (μM)[a]							
	**CYP isoform**							
	**1A2**	**2B6**	**2C8**	**2C9**	**2C19**	**2D6**	**3A4/5**[b]	**3A4/5**[c]
**Control**[d]	3.3	8.9	2.3	0.38	0.45	0.030	0.010	0.011
WEHI-P8	>20[e]	>20[e]	>20[e]	>20 (26%)	>20[e]	13.8	>20 (28%)	>20 (19%)

[a] Where less than 50% inhibition was observed at 20 μM (i.e. the highest concentration tested), the IC$_{50}$ value is deemed to be >20 μM and the % inhibition observed at this concentration is indicated in parentheses.

[b] midazolam 1'-hydroxylation

[c] testosterone 6β-hydroxylation

[d] Positive control IC$_{50}$ values were comparable with in-house historical data. Compounds used included: furafylline (CYP1A2), 2-phenyl-2-(1-piperidinyl) propane (CYP2B6), quercitin (CYP2C8), sulfaphenazole (CYP2C9), (S)-(+)-N-3-benzylnirvanol) (CYP2C19), quinidine (CYP2D6), ketoconazole (CYP3A4/5).

[e] No measurable inhibition observed with CYP activity in the presence of ***WEHI-P8***.

Time dependent CYP3A4/5 inhibition

CYP isoform	Inhibitor	Reference	IC_{50} (μM)[a]		Fold Shift[b]
			- NADPH	+ NADPH	
3A4/5[c]	Verapamil	Mechanism based	18.8	0.39	47.7
	Ketoconazole	Reversible	0.013	0.016	0.80
	WEHI-P8		>20 (25%)	>20 (30%)	c.n.c
3A4/5[d]	Verapamil	Mechanism based	9.5	0.24	40.3
	Ketoconazole	Reversible	0.0084	0.012	0.68
	WEHI-P8		>20 (37%)	>20 (36%)	c.n.c

[a] Where less than 50% inhibition was observed at 20 μM (i.e. the highest concentration tested), the IC_{50} value is deemed to be >20 μM and the % inhibition observed at this concentration is indicated in parentheses.

[b] Calculated as IC_{50} (-NADPH) / IC_{50} (+NADPH)

[c] midazolam 1’-hydroxylation

[d] testosterone 6β-hydroxylation

c.n.c. - Could not calculate as the IC_{50} was above the maximum concentration tested under both pre-incubation conditions.

Mouse exposure parameters

**Compound (nominal dose)**	**C_{max} (µM)**	**T_{max} (h)**	**Apparent $t_{1/2}$ (h)**	**$AUC_{0\text{-}last}$ (h*µM)**	**$AUC_{0\text{-}inf}$ (h*µM)**
WEHI-P8 (100 mg/kg)	7.07	1.0	14[a]	72.3	108[b]

[a] The terminal eliminaiton phase has been estimated on the basis of the last two time points only and therefore the apparent $t_{1/2}$ value is an approximation only.

[b] Value is an approximation only as the extrapolated portion contributed significantly (>30%) to $AUC_{0\text{-}inf}$.

Flow Cytometry Gating Strategy

FSC-A and SSC-A were used to gate live cells. FSC-A and FSC-H were used to gate single cells. mRuby3 (YG610/20) and FRET (B610/20) were used to select FRET positive cells.

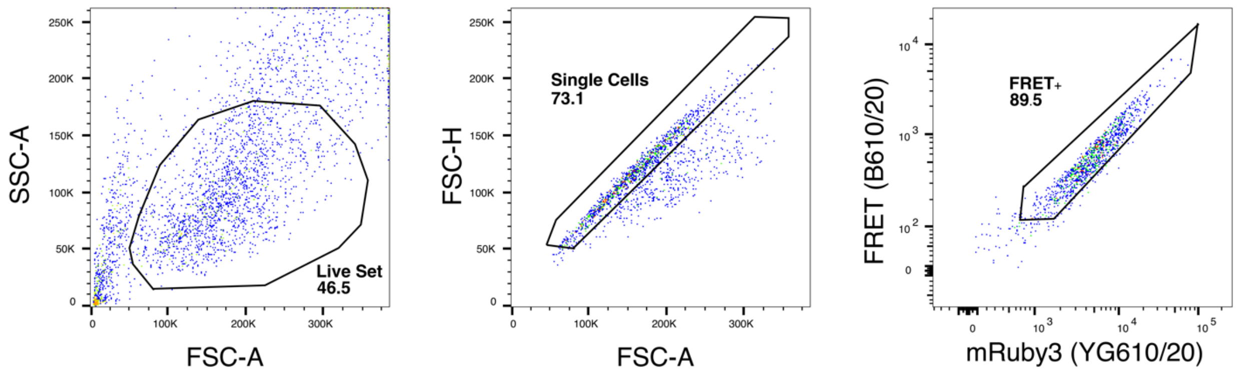

Supplementary References

1. Wu, X. *et al.* Mutational profiling of SARS-CoV-2 papain-like protease reveals requirements for function, structure, and drug escape. *Nat. Commun.* **15**, 6219 (2024).

2. Xiong, Y. *et al.* The substrate selectivity of papain-like proteases from human-infecting coronaviruses correlates with innate immune suppression. *Sci Signal* **16**, eade1985 (2023).

3. Garnsey, M. R. *et al.* Discovery of SARS-CoV-2 papain-like protease (PLpro) inhibitors with efficacy in a murine infection model. *Sci. Adv.* **10**, eado4288 (2024).

4. Bader, S. M. *et al.* SARS-CoV-2 mouse adaptation selects virulence mutations that cause TNF-driven age-dependent severe disease with human correlates. *Proc. Natl. Acad. Sci.* **120**, e2301689120 (2023).
