# Supplementary material for: A novel PLpro inhibitor improves outcomes in a pre-clinical model of long COVID
Source: Nat Commun. 2025 Apr 3;16:2900. doi: 10.1038/s41467-025-57905-4 (PMC11969009; doi:10.1038/s41467-025-57905-4)
Supplement: Supplementary file 2 — Reporting summary [file 41467_2025_57905_MOESM2_ESM.pdf]

nature portfolio

Corresponding author(s): David Komander, Marcel Doerflinger, Guillaume Lessene, Shane M. Devine

Last updated by author(s): Feb 28, 2025

Reporting Summary

Nature Portfolio wishes to improve the reproducibility of the work that we publish. This form provides structure for consistency and transparency in reporting. For further information on Nature Portfolio policies, see our Editorial Policies and the Editorial Policy Checklist.

Statistics

For all statistical analyses, confirm that the following items are present in the figure legend, table legend, main text, or Methods section.

n/a	Confirmed	
☐	☒	The exact sample size (*n*) for each experimental group/condition, given as a discrete number and unit of measurement
☐	☒	A statement on whether measurements were taken from distinct samples or whether the same sample was measured repeatedly
☐	☒	The statistical test(s) used AND whether they are one- or two-sided<br>*Only common tests should be described solely by name; describe more complex techniques in the Methods section.*
☒	☐	A description of all covariates tested
☐	☒	A description of any assumptions or corrections, such as tests of normality and adjustment for multiple comparisons
☐	☒	A full description of the statistical parameters including central tendency (e.g. means) or other basic estimates (e.g. regression coefficient) AND variation (e.g. standard deviation) or associated estimates of uncertainty (e.g. confidence intervals)
☐	☒	For null hypothesis testing, the test statistic (e.g. *F*, *t*, *r*) with confidence intervals, effect sizes, degrees of freedom and *P* value noted<br>*Give P values as exact values whenever suitable.*
☐	☒	For Bayesian analysis, information on the choice of priors and Markov chain Monte Carlo settings
☐	☒	For hierarchical and complex designs, identification of the appropriate level for tests and full reporting of outcomes
☐	☒	Estimates of effect sizes (e.g. Cohen's *d*, Pearson's *r*), indicating how they were calculated

Our web collection on statistics for biologists contains articles on many of the points above.

Software and code

Policy information about availability of computer code

Data collection

The following machines and software were used for data collection:

HTS/Screening: Tibco Spotfire v7.11.2, PHERAstar v5.41, BMG Labtech
SPR: Cytiva Biacore 8K Control Software v3.0.12.15655
Flow Cytometry data was collected with BD FACSDiva Software v9.1

Histology: VS200 scanner (Olympus).
Cytokines and chemokines: Bio-Plex 200 system (Bio-Rad)
Behaviour: CCTV Micro Surveillance Camera 700TVL CVBS (OuishiyuanTechnology)
OBS studio version 30.1.0
Proteomics: Orbitrap Astral (Thermo Fisher Scientific)

Data analysis

The following software and packages were used for data analysis:

Biacore Insight Evaluation Software v3.0.12.15655
Phenix v1.21rc1-5072-000
Coot 0.9.8.93
ChimeraX v1.7.1
Adobe Illustrator v28.6

Protein modelling and molecular dynamics were performed using the Schrödinger suite (Release 2024-2: Maestro, Schrödinger, LLC, New York, NY, 2024)
Flow Cytometry data: FlowJo v10.9.0
Spectronaut v19
GraphPad Prism (v10.2.3) GraphPad Software Schneider
RStudio (v1.4.1743-4, R 4.2.0) - R packages: ggplot2, ggpubr, openxlsx, reshape2
TopScan LITE (Cleversys Inc.)

For further details please see methods section.

For manuscripts utilizing custom algorithms or software that are central to the research but not yet described in published literature, software must be made available to editors and reviewers. We strongly encourage code deposition in a community repository (e.g. GitHub). See the Nature Portfolio guidelines for submitting code & software for further information.

Data

Policy information about availability of data

All manuscripts must include a data availability statement. This statement should provide the following information, where applicable:

- Accession codes, unique identifiers, or web links for publicly available datasets
- A description of any restrictions on data availability
- For clinical datasets or third party data, please ensure that the statement adheres to our policy

The mass spectrometry proteomics data have been deposited to the ProteomeXchange Consortium via the PRIDE partner repository with the dataset identifier PXD054356

Crystal structures:
The coordinates and crystallographic structure factors for SARS-CoV-2 PLpro in complex with WEHI-P1 have been deposited at the PDB under accession code 9CYB.
The coordinates and crystallographic structure factors for SARS-CoV-2 PLpro in complex with WEHI-P2 have been deposited at the PDB under accession code 9CYC.
The coordinates and crystallographic structure factors for SARS-CoV-2 PLpro in complex with WEHI-P4 have been deposited at the PDB under accession code 9CYD.
The coordinates and crystallographic structure factors for SARS-CoV-2 PLpro in complex with WEHI-P24 have been deposited at the PDB under accession code 9CYK.

Research involving human participants, their data, or biological material

Policy information about studies with human participants or human data. See also policy information about sex, gender (identity/presentation), and sexual orientation and race, ethnicity and racism.

Reporting on sex and gender	N/A
Reporting on race, ethnicity, or other socially relevant groupings	N/A
Population characteristics	N/A
Recruitment	N/A
Ethics oversight	N/A

Note that full information on the approval of the study protocol must also be provided in the manuscript.

Field-specific reporting

Please select the one below that is the best fit for your research. If you are not sure, read the appropriate sections before making your selection.

☒ Life sciences ☐ Behavioural & social sciences ☐ Ecological, evolutionary & environmental sciences

For a reference copy of the document with all sections, see nature.com/documents/nr-reporting-summary-flat.pdf

Life sciences study design

All studies must disclose on these points even when the disclosure is negative.

Sample size	For in vivo experiments, in accordance with the 3Rs, the smallest sample size was chosen that could give a significant difference (less than 0.05 type 1 error probability at 0.8 power). Pilot experiments were used to estimate the sample size such that an appropriate statistical test could yield significant results. The exact n numbers used in each experiment are indicated in the figure legends.<br>Sample sized for experiments involving drug treatments varied depending on compound availability.
Data exclusions	For behavior studies, data was excluded if an external factor disturbed animals during recording (such as unforeseen loud noise).<br>In quantification of histological gut sections, data points were excluded if processing of guts lead to mechanical damage.

Replication	Mouse experimental data from long COVID animals were repeated several times. Micrographs of histology are representative of at least three independent experiments. For long COVID experiments with previous drug treatment (Fig. 6), only one experimental cohort was analyzed.
Randomization	For mouse studies, mice were randomly allocated to infection/treatment groups.
Blinding	Pathologists were blinded during scoring of histological images, and researchers were blinded during microglia morphology quantification. Researchers were not blinded for treatment of mice to avoid mix-up of samples. Experimental design and appropriate controls ensured accuracy and reproducibility of measurements and analyses.

Reporting for specific materials, systems and methods

We require information from authors about some types of materials, experimental systems and methods used in many studies. Here, indicate whether each material, system or method listed is relevant to your study. If you are not sure if a list item applies to your research, read the appropriate section before selecting a response.

Materials & experimental systems

n/a	Involved in the study	
☐	☒	Antibodies
☐	☒	Eukaryotic cell lines
☒	☐	Palaeontology and archaeology
☐	☒	Animals and other organisms
☒	☐	Clinical data
☒	☐	Dual use research of concern
☒	☐	Plants

Methods

n/a	Involved in the study	
☒	☐	ChIP-seq
☐	☒	Flow cytometry
☒	☐	MRI-based neuroimaging

Antibodies

Antibodies used

Antibodies used for histology (manufacturer, cat number):
CD3 (Agilent A045201), MPO (Agilent A039829), F4/80 (WEHI in-house antibody) or SARS-CoV-2 nucleocapsid (abcam ab271180).

Validation

All antibodies listed in the previous section were validated by the manufacturer and/or by previous studies.

Information on the validation of antibodies for flow cytometry can be found as stated below:

Abcam: https://go.myabcam.com/BiophysicalQuality#:~:text=That's%20why%20we're%20continually,400%20added%20last%20year%20alone.
The high quality of our antibodies is founded on a range of precise validation techniques. Biophysical testing builds on these tools to let you know our antibodies in detail, so you can have confidence in your results no matter what kind of assay set-up you're using. They include: Recombinant technology, extensive application testing, advanced validation, knock-out validation.

Agilent: https://www.agilent.com/en/product/immunohistochemistry
Our immunohistochemistry products, including our IHC instruments, provide high staining quality, improved standardization and increased efficiency enabling the ultimate goal of increased patient safety. The broad portfolio of Agilent Dako products is continuously enhanced with new generations of carefully selected and clinically relevant reagents and instruments.

WEHI: In house- antibodies are validated through the WEHI histology facility. Different conditions of staining (buffers, dilutions, incubation conditions) are tested in initial optimization runs, including internal controls (tissue positive and negative) to establish antibody specificity and staining protocol.

Eukaryotic cell lines

Policy information about cell lines and Sex and Gender in Research

Cell line source(s)

We used authenticated HEK293T cells sourced from CellBank Australia, Cat# 12022001.
Mouse liver microsomes (pool of 1609 male CD1 mice) were sourced from XenoTech LLC, Kansas City, KS (lot #2210246).
Human liver microsomes (pool of 100 male and 100 female) were sourced from XenoTech LLC, Kansas City, KS (lot #1910096).
Mouse cryopreserved hepatocytes (pool of 16 male CD1 mice) were sourced from XenoTech LLC, Kansas City, KS (lot #2310051).
Human cryopreserved hepatocytes (pool 5 male and 5 female) were sourced from XenoTech LLC, Kansas City, KS (custom lot #2310092).
Mouse plasma (CD1, pooled, mixed gender, Na Heparin as anticoagulant) was sourced form BioIVT, Hicksville, NY (lot # MSE433327).
Human plasma (pooled, mixed gender, Na Heparin as anticoagulant) was sourced form BioIVT, Hicksville, NY (lot # HMN921520).
Vero cells: kidney epithelial cells from African green monkey.

Authentication	CellBank Australia supplies authenticated cell lines and morphology was consistent with the stated cell type.<br>Vero cells were purchased from ATCC (clone CCL-81) and used to make a cell bank. Vials are thawed and the cells were used until passage 30.
Mycoplasma contamination	All cell lines tested negative for mycoplasma contamination by PCR.
Commonly misidentified lines<br>(See ICLAC register)	No commonly misidentified cell lines were used in the study.

Animals and other research organisms

Policy information about studies involving animals; ARRIVE guidelines recommended for reporting animal research, and Sex and Gender in Research

Laboratory animals	WEHI studies: Male or female wild-type C57BL/6J, 9-14 week-old were used.<br>Monash University studies: Male or female wild-type C57BL/6, >7 weeks old were used.
Wild animals	No wild animals were used.
Reporting on sex	Both sexes were used, as direct comparison between SARS-CoV-2 P21 infected male and female C57BL/6 mice did not show any differences in viral loads (TCID50) or weight loss.
Field-collected samples	No field-collected samples were used.
Ethics oversight	Studies conducted at the Walter and Eliza Hall Institute: All procedures and mouse strains were reviewed and approved by The Walter and Eliza Hall Institute of Medical Research Animal Ethics Committee (ethics number 2020.016 and 2024.006) and were conducted in accordance with the Prevention of Cruelty to Animals Act (1986) and the Australian National Health and Medical Research Council Code of Practice for the Care and Use of Animals for Scientific Purposes (1997).<br><br>Studies at Monash University: Studies in mice at Monash University were conducted using established procedures in accordance with the Australian Code of Practice for the Care and Use of Animals for Scientific Purposes, and the study protocols were reviewed and approved by the Monash Institute of Pharmaceutical Sciences Animal Ethics Committee (ethics number 26789).

Note that full information on the approval of the study protocol must also be provided in the manuscript.

Plants

Seed stocks	N/A
Novel plant genotypes	N/A
Authentication	N/A

Flow Cytometry

Plots

Confirm that:

- [x] The axis labels state the marker and fluorochrome used (e.g. CD4-FITC).
- [x] The axis scales are clearly visible. Include numbers along axes only for bottom left plot of group (a 'group' is an analysis of identical markers).
- [x] All plots are contour plots with outliers or pseudocolor plots.
- [x] A numerical value for number of cells or percentage (with statistics) is provided.

Methodology

Sample preparation	HEK293T cells were lifted by pipeting, before proceeding to Flow Cytometry analysis or sorting.
Instrument	Flow Cytometry analysis was performed on BD FACSymphony A3 Cell Analyzer (BD Biosciences)
Software	BD FACSDiva software v9.1 was used for data collection; FlowJo 10.9.0 was used for data analysis.

Cell population abundance	We did not sort cells for the cellular assays. However, we ensured that around 2000 single cells were obtained during Flow Cytometry analysis, at which point the FRET percentage remained stable.
Gating strategy	FSC-A and SSC-A were used to gate live cells. FSC-A and FSC-H were used to gate single cell. mRuby3 (YG610/20) and FRET (B610/20) were used to select FRET positive cells.

☒ Tick this box to confirm that a figure exemplifying the gating strategy is provided in the Supplementary Information.
